# Multimodal deep learning fusion model for assessment of fetal lung development in gestational diabetes mellitus and pre-eclampsia

**DOI:** 10.3389/fendo.2026.1832468

**Published:** 2026-05-18

**Authors:** Yanran Du, Chao Ji, Jing Jiao, Fei Xin, Yunyun Ren, Zhenwei Xia, Yi Guo, Jianqiao Zhou

**Affiliations:** 1 Department of Ultrasound, Ruijin Hospital, Shanghai Jiaotong University School of Medicine, Shanghai, China; 2 Faculty of Medical Imaging Technology, College of Health Science and Technology, Shanghai Jiao Tong University School of Medicine, Shanghai, China; 3 Department of Anesthesiology, Ruijin Hospital, Shanghai Jiaotong University School of Medicine, Shanghai, China; 4 College of Biomedical Engineering, Fudan University, Shanghai, China; 5 Department of Pediatrics, Ruijin Hospital, Shanghai Jiaotong University School of Medicine, Shanghai, China; 6 Department of Ultrasound, Obstetrics and Gynecology Hospital of Fudan University, Shanghai, China

**Keywords:** deep learning, fetal lung development, gestational diabetes mellitus, multimodal, preeclampsia, ultrasound

## Abstract

**Background:**

Fetal lung development is highly sensitive to adverse intrauterine conditions such as gestational diabetes mellitus (GDM) and pre-eclampsia (PE). Current clinical evaluation mainly relies on ultrasound imaging, but it provides limited information on related histological and molecular changes. This study aimed to develop a multimodal deep learning framework that combined ultrasound imaging features with molecular and histopathological data to assess fetal lung development.

**Methods:**

Rat models of GDM and PE were established, and fetal lung ultrasound images were obtained. Fetal lung tissues were evaluated by histopathology. The expression of key proteins was analyzed by immunohistochemistry, Western blotting, and quantitative PCR. Gene sequencing was conducted, followed by differential expression and functional enrichment analyses. Deep learning algorithms were used for automated lung segmentation, quantitative feature extraction, and model development. By combining imaging features with molecular and histological data, a rat multimodal fusion model was constructed, which was then validated using human fetal lung ultrasound images through transfer learning and parameter optimization.

**Results:**

In animal studies, significant differences were observed in multiple indicators of fetal lung development among normal, GDM, and PE groups, including quantitative histopathology, immunohistochemical protein expression, qPCR results, gene sequencing profiles, and functional enrichment analysis. The performance of the multimodal fusion model was better than that of the ultrasound-only and partially integrated models, achieving accuracies of 0.935 (95% CI: 0.898, 0.973) and 0.948 (95% CI: 0.919, 0.970) and average AUC of 0.954 (95% CI: 0.919, 0.984) and 0.955 (95% CI: 0.932, 0.979) in mid- and late- gestation, respectively. In clinical studies, 1,183 images of human fetal lungs were analyzed, and the classification model based on transfer learning showed superior performance, with accuracies of 0.835 (95% CI: 0.786, 0.894) and 0.874 (95% CI: 0.828, 0.907) and average AUCs of 0.830 (95% CI: 0.772, 0.890) and 0.857 (95% CI: 0.824, 0.893) in early and late trimester pregnancy, respectively.

**Conclusions:**

This study demonstrated that integrating multimodal data improved the assessment of fetal lung development in GDM and PE. By linking imaging features with molecular and histopathological alterations, the proposed framework provides new methodological and biological insights and suggests a potential non-invasive strategy for monitoring fetal lung development in high-risk pregnancies.

## 1 Background

Fetal lung development is a strictly regulated and well-coordinated process, and it is essential for normal respiratory function after birth (1). It includes several morphological stages, starting from embryonic lung bud formation and continuing to alveolarization, and it is strongly influenced by the intrauterine environment. Pregnancy complications such as gestational diabetes mellitus (GDM) and pre-eclampsia (PE) can affect normal lung development and increase the risk of neonatal respiratory distress syndrome, bronchopulmonary dysplasia, and persistent pulmonary hypertension (2, 3).

Animal studies have shown that GDM and PE caused disease-specific changes in surfactant protein expression, angiogenic signaling, epithelial cell differentiation, and inflammatory pathways, and these changes may impair alveolar and vascular development (4, 5). In addition, in cases of fetal growth restriction or hypoxia, abnormal regulation of surfactant protein (SP) and vascular endothelial growth factor (VEGF) can limit alveolarization and capillary network formation (6, 7).

Conventional prenatal ultrasound has limited sensitivity and spatial resolution, so it is difficult to detect mild structural or functional changes in developing fetal lungs (8). However, when ultrasound imaging is combined with machine learning or deep learning (DL) methods, it can provide more detailed and non-invasive information on fetal lung development and allow real-time assessment (9–11). However, the imaging features obtained from fetal lung ultrasound still do not reflect the underlying molecular and cellular changes related to pregnancy-related disease. In addition, direct molecular or histopathological validation of human fetal lungs remains limited by ethical and technological constraints.

To bridge this gap, multi-level molecular analysis in animal models can provide biological references for imaging derived features. By integrating imaging data with molecular and structural information at multiple biological scales, these methods can improve diagnostic accuracy and deepen understanding of mechanisms (12–14). Although integrated imaging and multimodal data strategies have been widely explored in oncology for diagnosis and prognosis prediction (15–19), their application to fetal lung development is still limited.

In this study, we first developed a comprehensive framework that combined multimodal data (deep learning features of fetal rat lung ultrasound images, molecular and histopathological data) to evaluate fetal lung development during GDM, PE, and normal pregnancy. Then, transfer learning and parameter optimization methods were applied to verify the feasibility of the rat multimodal fusion model in more accurately evaluating fetal lung development using human fetal ultrasound images.

## 2 Methods

### 2.1 Animals and ethics

All animal procedures were approved by the Animal Ethics Committee of Ruijin Hospital (RJ2024016). Pregnant Wistar rats (11–12 weeks old, gestational day 13; Charles River Laboratories) were housed individually under standard conditions (22 ± 1 °C, 12:12-h light-dark cycle) with free access to food and water, and randomly assigned to three groups: normal, GDM, and PE.

GDM model: On gestational day 14, rats in the GDM group received a single intraperitoneal injection of streptozotocin (STZ, 45 mg/kg; Ibrox) (20). Control rats received sodium citrate buffer (pH 4.5). Fasting blood glucose levels were monitored on gestational days 16-18; levels >7.8 mmol/L confirmed successful GDM induction (21). Ten rats meeting these criteria were included.

PE model: PE was induced by tail vein infusion of lipopolysaccharide (LPS, 1.0 go/kg; Sigma-Aldrich) diluted in 1 mL saline on gestational day 14 using an infusion pump (1 mL/h) (22). Control rats received saline only. Systolic and diastolic blood pressures were measured on gestational days 18 and 21 using a noninvasive tail-cuff system (SciTech BP-2000). Urine samples were collected on gestational days 17 and 20 to assess albumin levels. Twelve rats with PE were included.

Sample collection: Cesarean sections were performed under 20% urethane anesthesia between gestational days 19 and 21. Blood samples were collected for liver and kidney function tests.

**Figure 1:**
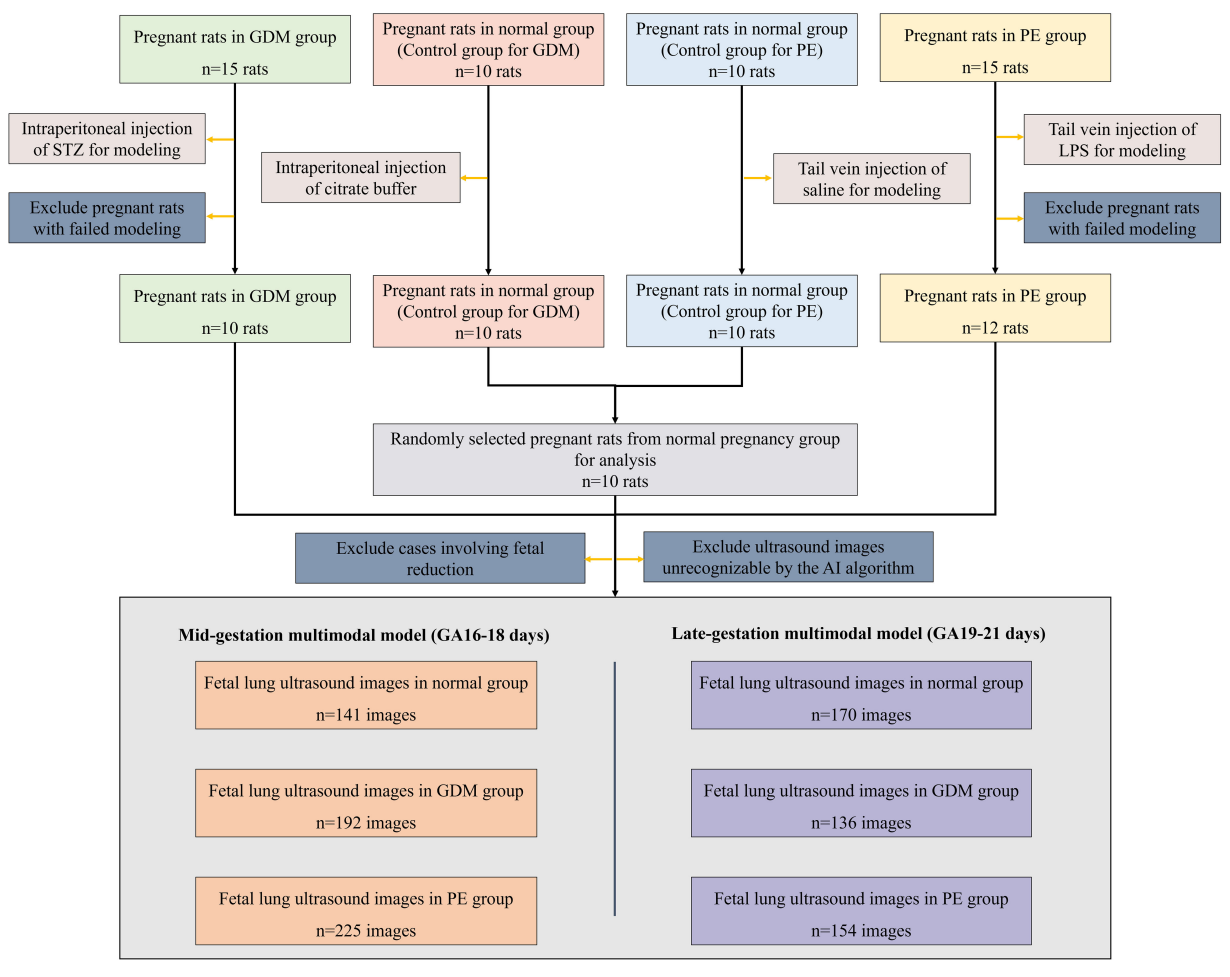
Flowchart of the selection of the rat population. GDM, Gestational Diabetes Mellitus; PE, Preeclampsia; STZ, Streptozotocin; LPS, Lipopolysaccharide; GA, Gestational Age.

To maintain group balance, samples from 10 normal pregnant rats were randomly selected for imaging and lung tissue analyses. The exclusion criteria were: 1) failed disease modeling, 2) unrecognizable ultrasound images or inaccurate region of interest (ROI) delineation, and 3) fetal reduction due to complications. The screening process for the rats is shown in **Figure 1**.

### 2.2 Patients and ethics

This was a retrospective study. Between July 2018 and October 2024, 2,253 routine fetal lung ultrasound images were collected from 2,253 women with singleton pregnancies. Gestational age (GA) ranged from 16^+3^ to 42^+0^ weeks. The data were collected at two centers, including the Obstetrics and Gynecology Hospital Affiliated to Fudan University and Ruijin Hospital, Shanghai Jiao Tong University School of Medicine. Written informed consent was obtained from all participants for the use of ultrasound images and clinical data. The study was approved by the ethics committees of both centers (2018–73 and 2023-429), and all procedures followed relevant guidelines and regulations.

**Figure 2:**
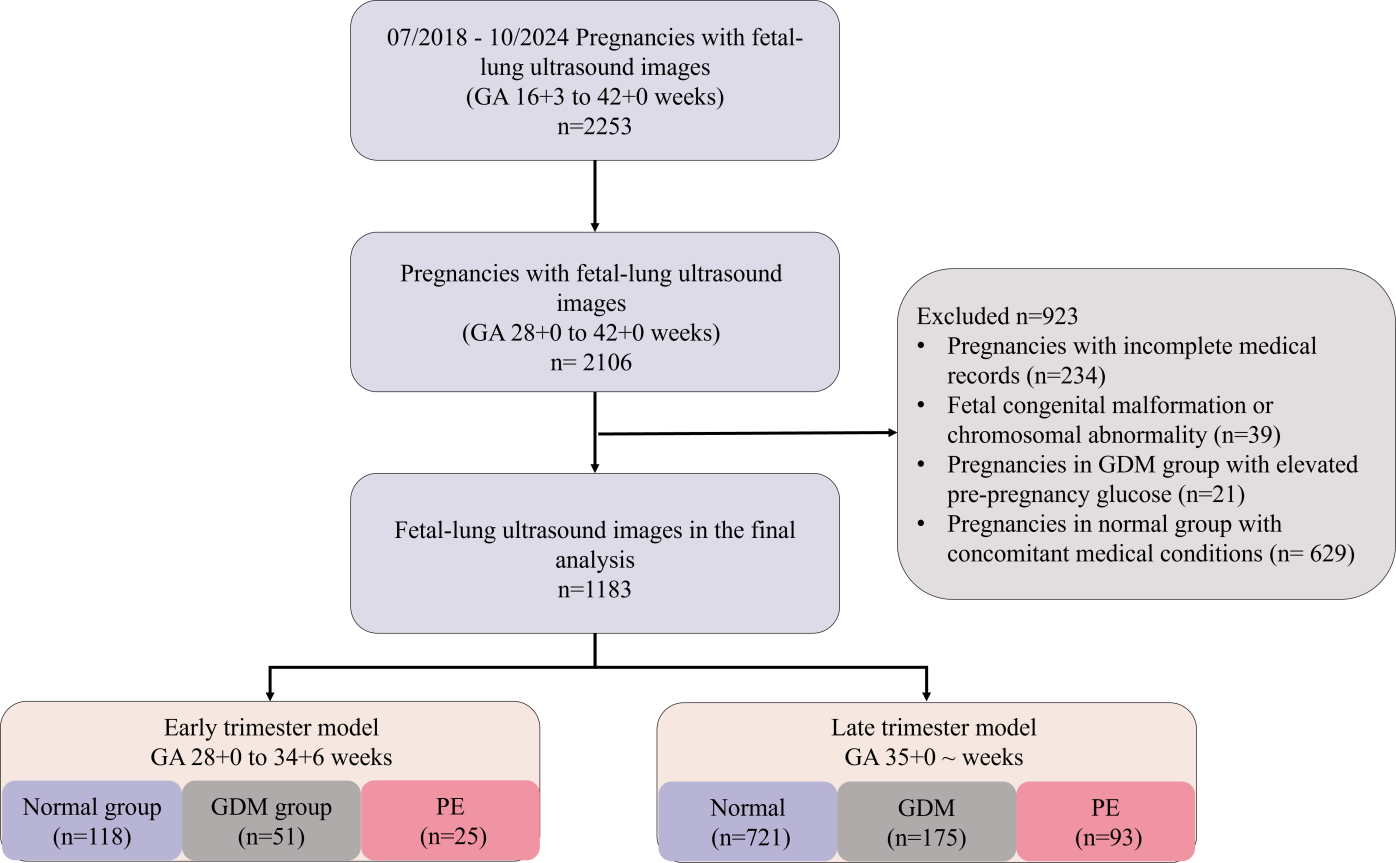
Flowchart of the selection of the human population. GA, Gestational Age; GDM, Gestational Diabetes Mellitus; PE, Preeclampsia.

A flowchart of the study population is shown in **Figure 2**. According to the enrollment criteria, the final cohort comprised 1,183 fetal-lung ultrasound images from 1,183 women. The enrollment criteria were: 1) Singleton pregnancy; 2) GA ≥28^+0^ weeks; 3) Pregnancies with complete medical records; 4) Fetuses without known congenital malformations or chromosomal abnormalities; 5) Pregnancies in the GDM group without pre-gestational diabetes; 6) Pregnancies in the normal group without any concomitant medical conditions. GA was calculated from the last menstrual period and confirmed by first-trimester crown-rump length measurements.

Pregnant women were categorized into GDM, PE, and normal groups. According to the GA at which fetal lung ultrasound images were captured, the women were further categorized into a 28^+0^ -34^+6^ weeks gestation (early) and 35^+0^ weeks gestation (late) group. GDM was diagnosed following the 2010 American Diabetes Association criteria (23) with a 75-g oral glucose tolerance test performed at 24–28 weeks; diagnostic thresholds were fasting glucose >5.1 mmol/L, 1-h >10.0 mmol/L, or 2-h >8.5 mmol/L. PE was diagnosed according to the 2014 International Society for the Study of Hypertension in Pregnancy criteria (24) as hypertension after 20 weeks of gestation (>140 mmHg systolic or >90 mmHg diastolic) with proteinuria (>300 mg/day) or evidence of maternal organ, uteroplacental, or fetal growth dysfunction.

### 2.3 Ultrasound imaging acquisition and ROI segmentation

#### 2.3.1 Ultrasound imaging acquisition

##### 2.3.1.1 Animal experimentation

Ultrasound examinations were performed using a portable M8 color Doppler system (Mindray, China) with an L20-5s probe set at 8–12 MHz. A senior radiologist (D.Y.R) with >15 years of experience in obstetric ultrasonography performed all scans.

Each pregnant rat underwent two ultrasound sessions: one during mid-gestation (GA 16–18 days) and the other during late-gestation (GA 19–21 days). Anesthesia was administered via intraperitoneal injection of 5% chloral hydrate (0.7 mg/100 g), followed by shaving of the abdominal fur and supine positioning. Ultrasound images of the fetal lungs were carefully acquired by gently maneuvering the fetuses.

Fetal lung images were acquired using a standardized protocol (25): (1) optimizing the transducer position to visualize at least one fetal lung; (2) adjusting imaging parameters (depth, gain, frequency, and harmonics) to suit individual conditions; and (3) excluding Doppler signals, annotations, or measurement markers.

For each fetus, the clearest lung image from each session was selected for analysis. All images were saved in DICOM (.dcm) format for offline processing.

##### 2.3.1.2 Human studies

All ultrasound images were obtained during routine prenatal ultrasound examinations. Image acquisition was performed by two radiologists with 10 and 5 years of experience in obstetric ultrasound. Two ultrasound systems were used: WS80A (Samsung, South Korea) equipped with a CA1-7A transducer (frequency range, 1–7 MHz; center frequency, 4.0 MHz), and VOLUSON E8 (General Electric Medical Group, USA) equipped with a C1-5-D transducer (frequency range, 2–5 MHz; center frequency, 3.5 MHz). The standardized image acquisition protocol has been previously described (9). Briefly, fetal lung images were obtained in an axial view of the thorax at the level of the four-chamber cardiac plane. Optimized system parameters, including depth, gain, frequency, and harmonic imaging, ensured that at least one lung had no rib-related acoustic shadows. All images were saved in DICOM (.dcm) format for offline processing.

#### 2.3.2 Automated segmentation of fetal lung regions

Using PyTorch 2.0 (Facebook Artificial Intelligence Institute, Menlo Park, California, USA), the automatic recognition of fetal lung ROIs was conducted based on the Medical Ultrasound Foundation Model (USFM) (26), which has been pretrained on a large-scale, multi-task, and multi-organ ultrasound dataset. The USFM provides high-quality feature extraction tailored for medical image-segmentation tasks.

Owing to the inherent differences between human and rat fetal lung images, a dataset of 600 normal fetal rat lung ultrasound images, each with manually annotated lung regions, was used to fine-tune the USFM. The segmentation network employed a Segmentation Vision Transformer head, a transformer-based module designed to enhance spatial feature representation. Subsequently, the fine-tuned model was applied to segment lung regions across the entire collection of fetal rat lung ultrasound images.

To ensure data consistency, all input images were preprocessed through grayscale normalization and resizing to achieve a uniform resolution. Standard data augmentation techniques, including random horizontal flipping, scaling, and rotation, were applied to improve the generalizability and robustness of the model.

During model optimization, a composite loss function was used, combining cross-entropy loss and Dice loss to effectively handle class imbalance and improve mask accuracy. The AdamW optimizer was used for parameter updates, with an initial learning rate of 1e-4. A cosine-annealing learning rate scheduler was used to progressively reduce the learning rate and facilitate faster convergence.

**Figure 3:**
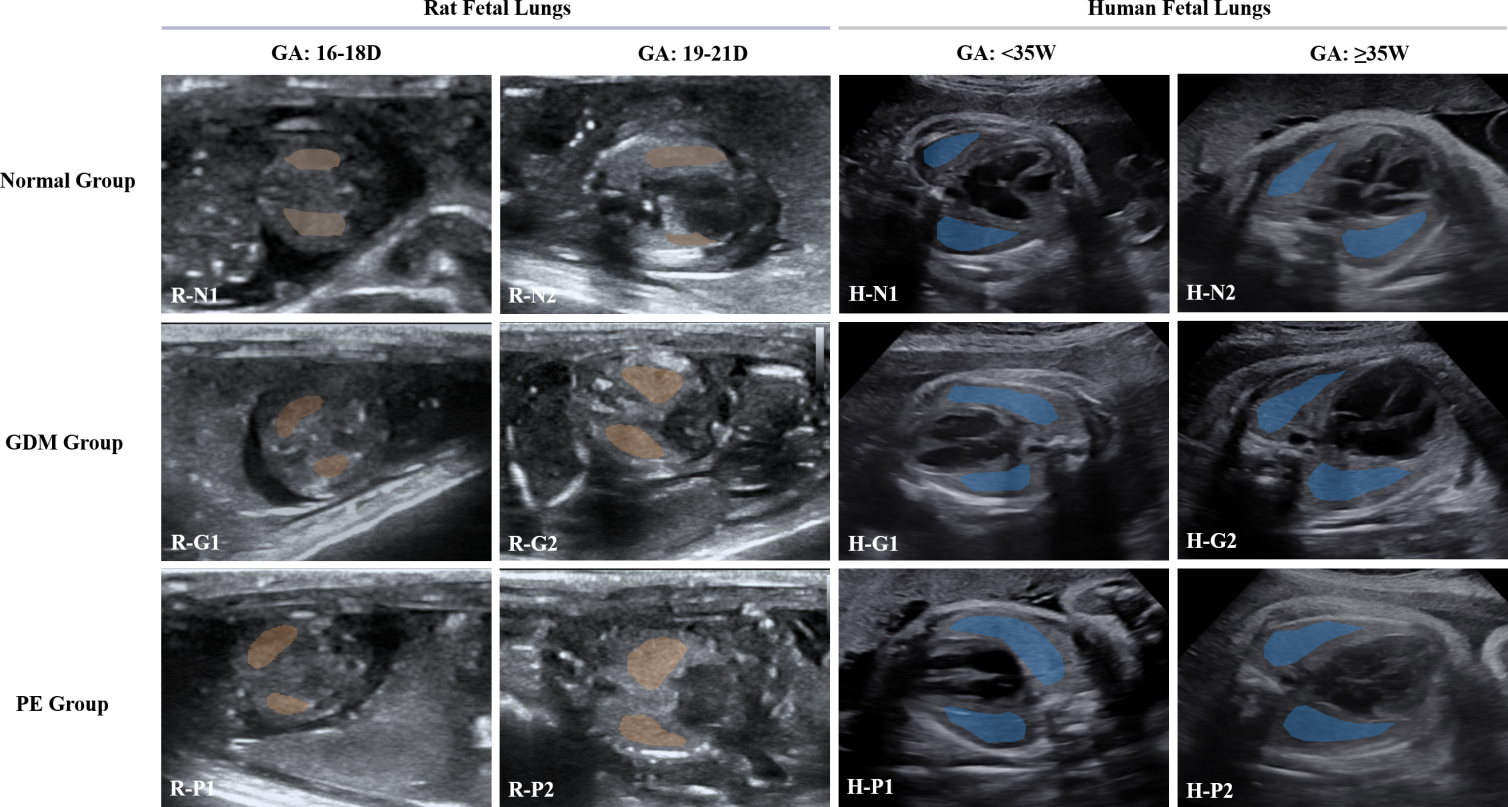
Fetal lung images with USFM-based automatic ROI recognition. GDM, Gestational Diabetes Mellitus; PE, Preeclampsia; USFM, Medical Ultrasound Foundation Model; ROI, Region of Interest. Rat fetal lungs: “R-N, G, P” denote the normal, GDM, and PE groups, respectively. N1, G1, and P1 were collected at a gestational age (GA) of 16–18 days; N2, G2, and P2 at a GA of 19–21 days. Human fetal lungs: “H-N, G, P” denote the normal, GDM, and PE groups, respectively. N1, G1, and P1 were collected at GA 28^+0^-34^+6^ weeks; N2, G2, and P2 at GA ≥35^+0^ weeks.

Representative fetal lung ultrasound images and automatically identified lung ROIs are shown in **Figure 3**.

### 2.4 Urinary protein and serum biochemical markers

The 24-h urinary protein concentration in pregnant rats was measured using an Albumin Assay Kit (Nanjing Jianchen, A028-2-1) per the manufacturer’s instructions. Liver and kidney functions in the PE and normal groups were assessed using commercial kits (Leidu/Changchun Huili). Liver function was evaluated using alanine aminotransferase and aspartate aminotransferase activities, whereas kidney function was assessed using urea, serum creatinine, blood urea nitrogen, and uric acid levels. Absorbance readings were obtained using a fully automated biochemical analyzer, and enzyme activity and metabolite concentrations were calculated from the standard curves.

### 2.5 Histopathology

Hematoxylin and eosin (HE)-stained lung sections were used for histological analyses. The embedded wax block of lung tissue was continuously sliced and cut into 5 μm‐thick pieces. Six sections of each lung tissue were selected at specific intervals for histopathological staging, performed by a lung pathologist in a blinded manner. Lung development is divided into five stages (27), including the embryonic (E9-11.5), pseudoglandular (E11.5-16.5), canalicular (E16.5-17.5), terminal sac (E17.5-P5), and alveolar (P5-P28) periods.

Histopathological images were measured and analyzed by a lung pathologist using ImageJ. Quantitative analysis results included developmental stage classification, alveolar area, septal thickness, airway wall thickness, airway diameter, and medial wall thickness (MWT) of pulmonary arteries.

### 2.6 Immunohistochemistry

IHC was performed on paraffin-embedded lung sections using standard protocols. After dewaxing, rehydration, and antigen retrieval in citrate buffer (pH 6.0), endogenous peroxidase activity was blocked with 3% H_2_O_2_, followed by blocking with 3% bovine serum albumin. Sections were incubated overnight at 4 °C with anti-SFTPD antibody (1:200, Thermo Fisher), then with horseradish peroxidase (HRP)-conjugated secondary antibody (Aifang Biotech). Signals were developed using 3,3’-Diaminobenzidine and counterstained with hematoxylin. The slides were dehydrated, mounted, and scanned.

Quantification was conducted using KFSlideOS and ImageJ. The mean optical density (OD) reflected the expression levels, and the percentage of positively stained area (%Area) represented the distribution. Data were averaged from ≥5 randomly selected fields per section under identical conditions.

### 2.7 Quantitative real-time polymerase chain reaction analysis

Total RNA was extracted using TRIzol™ reagent (Thermo Fisher Scientific) and quantified using a NanoDrop spectrophotometer. cDNA synthesis was performed using the Evo M-MLV Reverse Transcriptase Premix (Accurate Biology). qPCR was performed with SYBR^®^ Green Premix Pro Taq HS (Accurate Biology) on a QuantStudio™ 6 Flex system (Applied Biosystems). β-Actin served as the internal control, and relative gene expression was calculated using the 2^−ΔΔCt method. The primer sequences are listed in **Supplementary Table 1**.

### 2.8 Protein extraction and western blot analysis

Proteins were extracted using RIPA buffer (Beyotime) supplemented with protease and phosphatase inhibitors. After centrifugation at 12,000 rpm for 10 min at 4 °C, supernatants were collected and quantified using the bicinchoninic acid assay Protein Assay Kit (Thermo Fisher Scientific). Equal amounts of protein were resolved by 10–15% sodium dodecyl sulfate-polyacrylamide gel electrophoresis and transferred to polyvinylidene fluoride membranes (Millipore). Membranes were blocked with 5% non-fat milk for 90 min at room temperature, then incubated overnight at 4 °C with primary antibodies against SP-A, SP-B, SP-C, SP-D, VEGF, and β-actin. After incubation with HRP-conjugated secondary antibodies, signals were visualized using ECL (Millipore) and imaged using a Tanon 5200 system. All experiments were performed in triplicates.

### 2.9 RNA sequencing and analysis

#### 2.9.1 RNA isolation and library preparation

Total RNA was extracted using TRIzol reagent (Invitrogen) and assessed for purity and concentration using a NanoDrop 2000 spectrophotometer (Thermo Scientific). RNA integrity was confirmed using the Agilent 2100 Bioanalyzer. Libraries were prepared using the VAHTS Universal V10 RNA-seq Library Prep Kit and sequenced on the Illumina NovaSeq 6000 platform (OE Biotech, Shanghai, China), generating 150 bp paired-end reads.

#### 2.9.2 RNA sequencing and differentially expressed gene analysis

Sequencing libraries were constructed and sequenced on the Illumina NovaSeq 6000 platform to generate 150 bp paired-end reads. Each sample yielded ~21.21–25.88 million raw reads, filtered using fastp to remove low-quality reads and adapters (28), resulting in ~19.75–23.95 million clean reads per sample.

Clean reads were aligned to the reference genome using HISAT2 (29). Gene expression was quantified as FPKM (30), and read counts were obtained via HTSeq-count (31). DEGs were identified using DESeq2 (Q-value < 0.05; fold-change > 2 or < 0.5). Hierarchical clustering of the DEGs was performed using R (v3.2.0) to visualize group-specific expression patterns.

Based on the hypergeometric distribution, Gene Ontology (GO) and Kyoto Encyclopedia of Genes and Genomes (KEGG) pathway enrichment analyses of DEGs were performed to screen for significantly enriched terms using R software (version 3.2.0; R Foundation for Statistical Computing, Vienna, Austria). R software was used to draw the chord and bubble diagrams of the significant enrichment terms.

#### 2.9.3 DEG identification and selection

To identify candidate genes with significant and overlapping expression changes, differential expression analysis was performed for three pairwise comparisons: GDM vs. control, PE vs. control, and GDM vs. PE. For each comparison, the top 10 upregulated and downregulated genes were selected (based on the fold change and false discovery rate < 0.05). Genes differentially expressed in at least two of the three comparisons were retained, resulting in 12 DEGs for further analysis, potentially implicated in the shared pathogenic pathways of GDM and PE: *Hgd*, *Clec4e*, *Nkx3-1*, *Kiss1*, *Cnmd*, *Hbz*, *Fga*, *Krt13*, *Cxcl2*, *Col2a1*, *Pax1*, and *Dlx3*.

### 2.10 Establishment of classification model for fetal lung development in pregnancy complications

**Figure 4:**
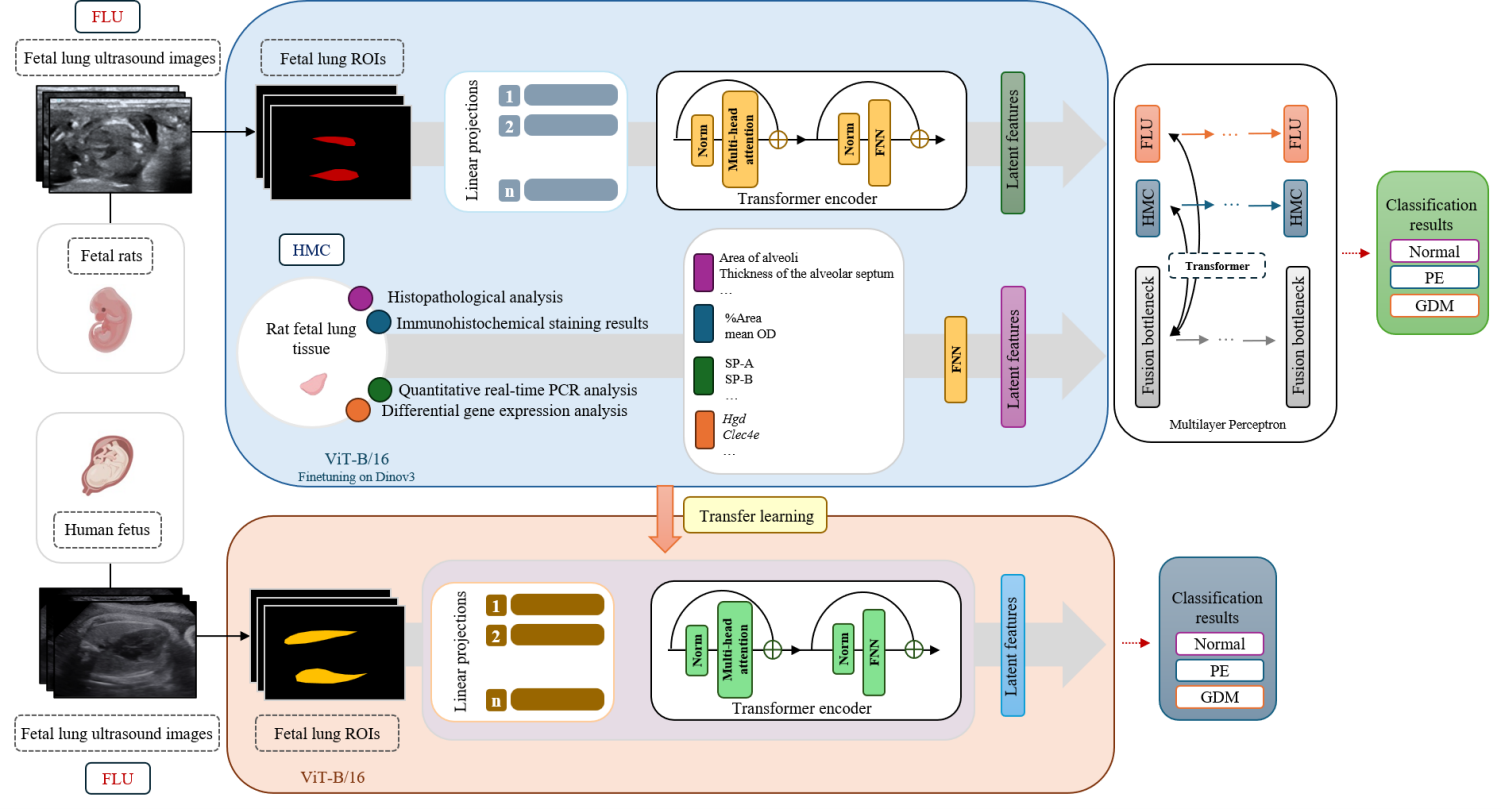
Workflow for developing a deep learning-based classification model for fetal lung development in pregnancy complications. GDM, Gestational Diabetes Mellitus; PE, Preeclampsia; ROI, Region of Interest; FLU, Fetal Lung Ultrasound; HMC, Histology Molecular Characterization; MLP, Multilayer Perceptron. Upper panel (rat model): Fetal lung ultrasound images from pregnant rats were preprocessed with automated ROI extraction and subsequently encoded using a Dino v3 fine-tuned ViT-B/16 framework. Parallel histopathological evaluation, quantitative immunohistochemistry, qRT-PCR, and differential gene expression profiling of fetal lung tissues yielded complementary molecular and cellular characteristics. Integrated latent imaging molecular features were classified using a multilayer perceptron to differentiate the fetal lung status under normal, PE, and GDM conditions. Lower panel (human model): For the human data, transfer learning was implemented using a rat multilevel model. Automatically extracted human fetal lung ROIs underwent the same ViT-B/16 encoding with parameter adaptation, enabling the classification of fetal lung development across normal, PE, and GDM pregnancies.

The workflow for establishing a classification model of human fetal lung development during pregnancy complications is shown in **Figure 4**. All feature extraction and model development were performed using PyTorch 2.0 (Facebook Artificial Intelligence Institute, Menlo Park, California, USA). In this study, features were learned automatically by the deep learning model. The model generated deep feature representations from intermediate network layers.

Owing to differences in lung development at different GAs, rat and human fetal samples were segmented based on GAs for feature extraction and model construction. Fetal rat samples were classified into two groups: the mid- (GA 16–18 days) and late- (GA 19–21 days) gestation models. Human fetal samples were categorized into two groups: the early (GA 28^+0^ - 34^+6^ weeks) and late (GA ≥35^+0^ weeks) trimester pregnancy groups. The methods of feature extraction, selection, and modeling were consistent for both sample models.

To evaluate the baseline model performance on the rat dataset, the classification ability was first assessed using only ultrasound images. To further explore the biological characteristics of fetal lung development, our analysis was extended by combining additional biological features with deep learning features from fetal lung ultrasound images. Pathological features (quantitative histopathological results of fetal rat lung tissue), immunohistochemical data (immunohistochemical quantitative analysis results of key proteins), molecular features (qPCR expression levels of key proteins), and genetic features (top differentially expressed gene data) were included.

Various mixed models were classified according to the type of biological information integrated as follows:

1. Ultrasound-based model (US): A classification model that evaluated fetal lung development based solely on deep learning features extracted from ultrasound imaging.
2. Ultrasound + Pathology & IHC model (US + Path & IHC): A model that integrated ultrasound image features with histopathological analysis and immunohistochemical data.
3. Ultrasound + qPCR model (US + qPCR): A model that combined ultrasound image features with qPCR data.
4. Ultrasound + gene sequencing model (US+ Seq): A model that integrated ultrasound image features with transcriptomic information derived from the top DEGs identified by gene sequencing.
5. Fully integrated multimodal data model (Multimodal): A fully integrated model that combined ultrasound image features with histopathology, IHC, qPCR, and gene sequencing data.

These features were incorporated into the fine-tuned ViT-B/16 architecture. The backbone network was initialized using the weights of Dino v3 (Self-Distillation with No Labels v3) (32), a large-scale self-supervised vision foundation model developed by Meta AI. It was pretrained on approximately 1.69 billion images using a Vision Transformer backbone, enabling the extraction of highly generalizable structural and textural representations without requiring manual annotations. Fine tuning the rat dataset enabled the model to adapt these pre-trained representations to the specific acoustic features and morphological patterns of rat fetal lungs. The predictive performance of various combination models was evaluated using accuracy, sensitivity, specificity, positive predictive value (PPV), negative predictive value (NPV), F1 score, and the area under the receiver operating characteristic curve (AUC) for each group, as well as their average values.

After that, a cross-species analysis was performed. The multimodal rat model was used to fine-tune the feature encoder of human fetal lung ultrasound images through a transfer learning framework. This approach allowed the model to adjust to species-related differences in grayscale distribution and texture patterns, and it improved the extraction of imaging features related to human fetal lungs. Model performance was then compared with that of a model trained only on human fetal lung ultrasound images. Predictive performance was also assessed using accuracy, sensitivity, specificity, PPV, NPV, F1 score, and AUC.

### 2.11 Statistical analysis

Continuous variables were analyzed using the Student’s t-test or one-way analysis of variance. Categorical variables were analyzed using the Pearson chi-square test or Fisher’s exact test, based on sample size. A *P* value of less than 0.05 was considered statistically significant. All analyses were performed using SPSS version 29.0.2.0 (IBM Corp., Armonk, NY, USA). Statistical methods for artificial intelligence models were described in the relevant sections.

## 3 Results

### 3.1 Overview of study design and research cohorts

**Figure 5:**
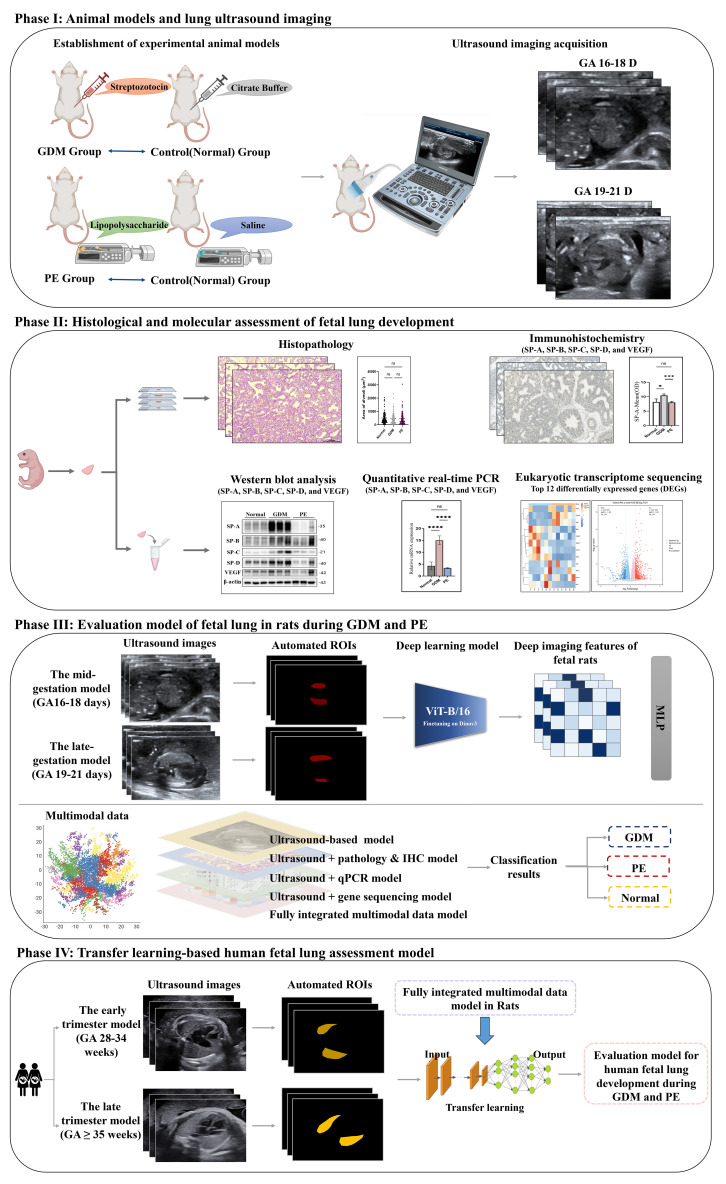
Schematic of the study. Framework of the Four-Phase Study. GDM, Gestational Diabetes Mellitus; PE, Preeclampsia; GA, Gestational Age; SP, Surfactant Protein; VEGF, Vascular Endothelial Growth Factor; ROI, Region of Interest. IHC, Immunohistochemistry.

This study aimed to evaluate fetal lung development at different GAs of pregnancy using an ultrasound-based deep learning approach. The workflow comprised four sequential phases (**Figure 5**). In the first phase, three rat models were established: GDM, PE, and normal controls. Fetal lung ultrasound images were acquired during mid- and late-gestation. In the second phase, fetal lung tissues were subjected to histopathological and molecular analyses, including qualitative and quantitative assessments, immunohistochemical staining, WB, and qPCR for SP-A, SP-B, SP-C, SP-D, and VEGF expression, as well as RNA sequencing to identify the top 12 DEGs across the three groups. In the third stage, the deep learning features derived from ultrasound images were combined with histological and molecular data, and the diagnostic performance of various combination models were compared separately. Finally, using transfer learning techniques, the rat fetal lung classification model integrated with multimodal data was validated using human fetal lung ultrasound images, and parameters were optimized to establish a classification model for evaluating human fetal lung development during pregnancy complications.

#### 3.1.1 Animal cohort

558 fetal lung ultrasound images were included in the mid-gestation model (GA 16–18 days): normal (n = 141), GDM (n = 192), and PE (n = 225). For the late-gestation model (GA 19–21 days), 460 images were included: normal (n = 170), GDM (n = 136), and PE (n = 154). A total of 460 fetal lung tissue samples from 460 fetuses were ultimately included in the analysis, comprising 170, 136, and 154 from the normal, GDM, and PE groups, respectively. A comparative analysis of fetal body and lung weights among the three groups is presented in **Table 1**.

**Table 1:** Comparison of the weights of newborn rats and fetal lungs among three groups.

Weight	Normal group	GDM group	PE group	P value
Newborn rats	0.108-4.379 (3.441 ± 1.007)	0.556-6.362 (4.524 ± 1.309)	0.048-5.800 (2.741 ± 0.944)	*P^1^* = 0.001* P^2^ = 0.002* P^3^ < 0.001*
Fetal lungs	23.47-127.60 (101.32 ± 23.47)	8.50-136.90 (74.98 ± 37.28)	21.74-136.80 (80.98 ± 21.74)	*P^1^* = 0.001* P^2^ = 0.000* P^3^ = 0.292

GDM, Gestational Diabetes Mellitus; PE, Preeclampsia; *P*^1^ value is the comparison between the normal and GDM groups, *P*^2^ value is the comparison between the normal and PE groups, and *P*^3^ value is the comparison between the GDM and PE groups. ^*^ *P* < 0.05 indicates a significant difference.

#### 3.1.2 Human cohort

The study included 1,183 fetal lung ultrasound images, including 839 (70.92%), 226 (19.10%), and 118 (9.97%) in the normal, GDM, and PE groups. The mean ± standard deviation age of the pregnant women was 31 ± 3.97 years. According to gestational age at fetal-lung ultrasound imaging, there were 194 (16.40%) cases in the 28^+0^-34^+6^ weeks group and 989 (83.60%) cases in the ≥35^+0^ weeks group. In the 28^+0^-34^+6^ weeks group, there were 118 (60.82%) cases in the normal group, 51 (26.29%) cases in the GDM group, and 25 (12.89%) cases in the PE group. In the ≥35^+0^ weeks group, there were 721 (72.90%) cases in the normal group, 175 (17.69%) cases in the GDM group, and 93 (9.41%) cases in the PE group. The characteristics of the human cohort are summarized in **Table 2**.

**Table 2:** Characteristics of the human cohort.

Characteristic	Normal group (n=839)	GDM group (n=226)	PE group (n=118)	Total (n=1183)
Maternal age (years)	31 ± 3.93	32 ± 4.15	31 ± 3.62	31 ± 3.97
Gestational age at ultrasound				
28 + 0-34 + 6 weeks	118 (14.06)	51 (22.57)	25 (21.19)	194 (16.40)
≥35 + 0 week	721 (85.94)	175 (77.43)	93 (78.81)	989 (83.60)
Mode of delivery				
Spontaneous vaginal	520 (61.98)	137 (60.62)	55 (46.61)	712 (60.19)
Cesarean	319 (38.02)	89 (39.38)	63 (53.39)	471 (39.81)
Birth weight (g)	3397 ± 377	3309 ± 399	3189 ± 608	3406 ± 392
Sex of newborn				
Female	394 (46.96)	115 (50.88)	60 (50.85)	569 (48.10)
Male	445 (53.04)	111 (49.12)	58 (49.15)	614 (51.90)
5-min Apgar score				
≤7	4 (0.48)	3 (1.33)	2 (1.69)	9 (0.76)
>7	835 (99.52)	223 (98.67)	116 (98.31)	1174 (99.24)
Neonatal prognosis				
No neonatal respiratory morbidity	819 (97.62)	217 (96.02)	102 (86.44)	1138 (96.20)
Neonatal respiratory morbidity	20 (2.38)	9 (3.98)	16 (13.56)	45 (3.80)

GDM, Gestational Diabetes Mellitus; PE, Preeclampsia; SD, Standard Deviation; Data are presented as means ± SDs or n (%).

### 3.2 Histological and molecular assessment of fetal lung development

#### 3.2.1 Pathological findings

**Figure 6:**
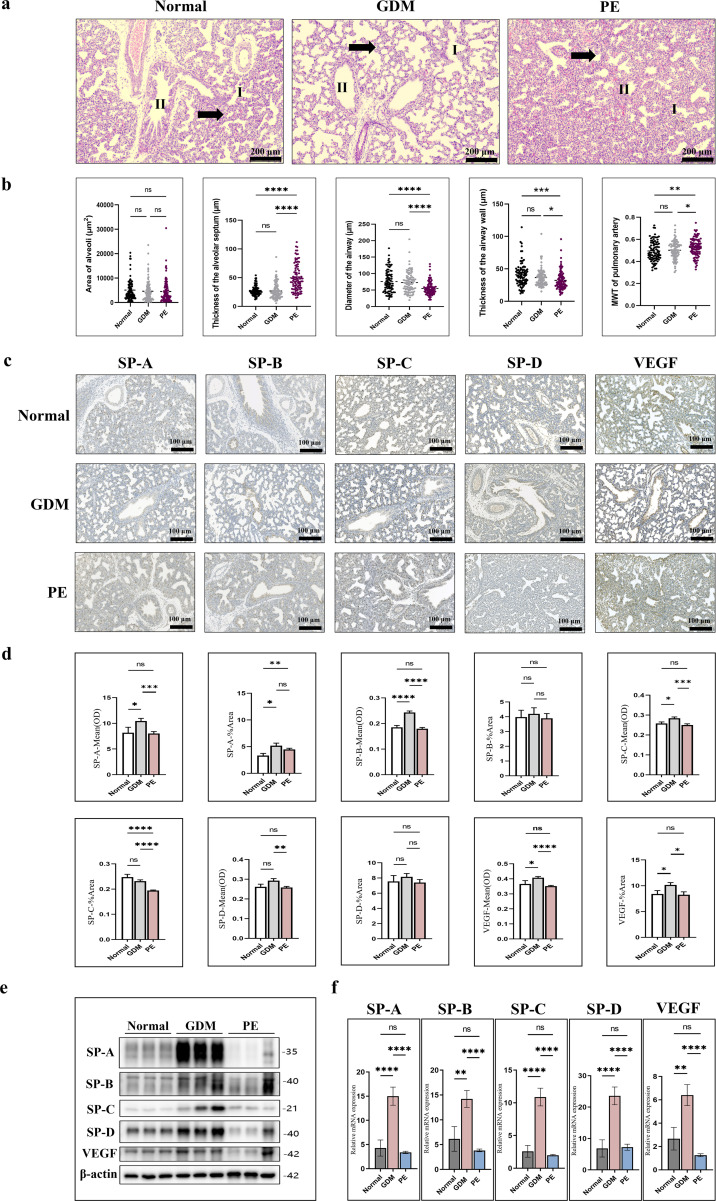
Histopathological and molecular biology results of fetal lung tissue in normal, GDM, and PE groups. MWT, Medial Wall Thickness; GDM, Gestational **(a)** Representative H&E-stained fetal lung sections from normal, GDM, and PE groups (×100; scale bar = 200 μm). Alveoli (I) and bronchioles (II) are indicated; black arrows denote the alveolar septa. Normal lungs show well-expanded alveoli with thin septa and intact airways. GDM lungs exhibit uneven septal thickness and irregular bronchial walls. PE lungs show reduced irregular alveoli, marked septal thickening, and narrowed thick-walled airways. **(b)** Quantitative histological analysis: (D) area of the alveoli, (E) thickness of the alveolar septum, (F) diameter of the airway, (G) thickness of the airway wall, and (H) MWT of the pulmonary artery. Data are shown as mean ± SEM. **(c)** Representative immunohistochemical staining for SP-A, SP-B, SP-C, SP-D, and VEGF (×200; scale bar = 100 μm). Brown staining indicates positive expression, mainly in alveolar and airway epithelial cells. **(d)** Quantification of immunohistochemical staining, expressed as mean optical density (OD) and percentage of positive areas. Data are expressed as mean ± SD. **(e) **Representative Western blot images showing protein expression of SP-A, SP-B, SP-C, SP-D, and VEGF in fetal lung tissues. β-actin served as the loading control. **(f)** Relative mRNA expression of SP-A, SP-B, SP-C, SP-D, and VEGF determined by qPCR. Data are expressed as mean ± SD. Statistical significance is indicated as *P < 0.05; **P < 0.01; ***P < 0.001; ****P < 0.0001; ns, not significant.

To assess the developmental status and structural changes in the fetal lungs across the three groups, we conducted a quantitative histopathological analysis, which included parameters of alveolar structure, airway structure, and pulmonary vasculature. The results are presented in **Table 3**; **Figures 6A, B**.

**Table 3:** Histopathological characteristics of rat fetal lungs.

Characteristics	Normal group	GDM group	PE group	*P* value
Area of alveoli (μm^2^)	471.78-20387.36 (5077.56 ± 4198.02)	255.21-23533.50 (4604.85 ± 4141.44)	252.88-30499.30 (4522.55 ± 4470.13)	*P*^1^ = 0.436, *P*^2^ = 0.380, *P*^3^ = 0.893
Thickness of the alveolar septum (μm)	12.20-54.24 (27.94 ± 8.75)	5.68-86.11 (26.89 ± 13.17)	14.88-112.02 (49.63 ± 22.98)	*P*^1^ = 0.524, *P*^2^, *P*^3^<0.001^*^
Thickness of the airway wall (μm)	12.01-114.21 (40.99 ± 18.31)	15.20-103.96 (36.54 ± 13.22)	9.95-95.85 (32.15 ± 14.64)	*P*^1^ = 0.054, *P*^2^ = 0.000^*^, *P*^3^ = 0.027^*^
Diameter of the airway (μm)	22.85-177.05 (75.88 ± 33.67)	19.65-204.34 (72.82 ± 30.37)	20.53-129.07 (54.92 ± 19.91)	*P*^1^ = 0.511, *P*^2^, *P*^3^<0.001^*^
MWT of pulmonary artery	0.320-0.726 (0.495 ± 0.090)	0.303-0.727 (0.501 ± 0.079)	0.328-0.750 (0.528 ± 0.081)	*P*^1^ = 0.604, *P*^2^ = 0.009^*^, *P*^3^ = 0.020^*^

MWT, Medial Wall Thickness; GDM, Gestational Diabetes Mellitus; PE, Preeclampsia.

*P*^1^ value is the comparison between the normal and GDM groups, *P*^2^ value is the comparison between the normal and PE groups, and *P*^3^ value is the comparison between the GDM and PE groups. ^*^ *P* < 0.05 indicates a significant difference.

Histopathological analysis of fetal lung tissues revealed no significant differences between the normal and GDM groups (all *P* > 0.05). However, all parameters except alveolar surface area showed significant differences between the PE group and both the normal and GDM groups. Compared with the normal and GDM groups, the PE group exhibited a significantly increased alveolar septal thickness (49.63 ± 22.98 µm [PE group] vs. 27.94 ± 8.75 µm [normal group] and 26.89 ± 13.17 µm [GDM group]), while the airway wall thickness and the airway diameter was significantly decreased (airway wall thickness: 32.15 ± 14.64 µm [PE group] vs. 40.99 ± 18.31 µm [normal group] and 36.34 ± 15.13 µm [GDM group]; airway diameter: 54.92 ± 19.91 µm [PE group] vs. 75.88 ± 33.67 µm [normal group] and 72.82 ± 30.37 µm [GDM group]) (all *P* < 0.05).

Regarding pulmonary vasculature, the MWT of the pulmonary artery was significantly increased in the PE group (0.528 ± 0.081) compared with the normal (0.495 ± 0.090) and GDM groups (0.501 ± 0.079) (both *P* < 0.05).

#### 3.2.2 Immunohistochemical findings

Immunohistochemical analysis was performed to assess the expression of SP-A, SP-B, SP-C, SP-D, and VEGF in fetal lung tissues across the normal, GDM, and PE groups (**Supplementary Table 2**; **Figures 6C, D**). The %Area of SP-A was significantly increased in both the GDM and PE groups compared with that in the normal group (*P* < 0.05), whereas its mean OD was significantly lower in the PE group (*P* < 0.05). SP-B mean OD was elevated in the GDM group (*P* < 0.05), with no significant differences in %Area among the groups. SP-C expression (both mean OD and %Area) was significantly higher in the GDM group than in the normal and PE groups (*P* < 0.05), with no significant changes in the PE group. SP-D levels showed no significant differences among the three groups. VEGF expression (mean OD and %Area) was significantly higher in the GDM group than in the normal and PE groups (*P* < 0.05), with no significant differences between the PE and normal groups (*P* > 0.05).

#### 3.2.3 WB and qPCR analysis

WB and qPCR were conducted to assess the expression of SP-A, SP-B, SP-C, SP-D, and VEGF in fetal lung tissues from the normal, GDM, and PE groups. WB analysis (**Figure 6E**) showed significantly increased protein levels of all five markers in the GDM group compared with those in the normal group (*P* < 0.05), with no significant changes observed in the PE group (*P* > 0.05).

qPCR results (**Supplementary Table 3**; **Figure 6F**) were consistent with the protein level findings. mRNA expression of SP-A, SP-B, SP-C, SP-D, and VEGF was significantly higher in the GDM group than in the normal and PE groups (*P* < 0.05). In the PE group, mRNA levels of SP-A, SP-B, SP-C, and VEGF were slightly decreased, whereas those of SP-D were slightly increased, although the difference was not significant (*P* > 0.05).

### 3.3 Representative DEGs and functional annotation

#### 3.3.1 DEGs

**Figure 7:**
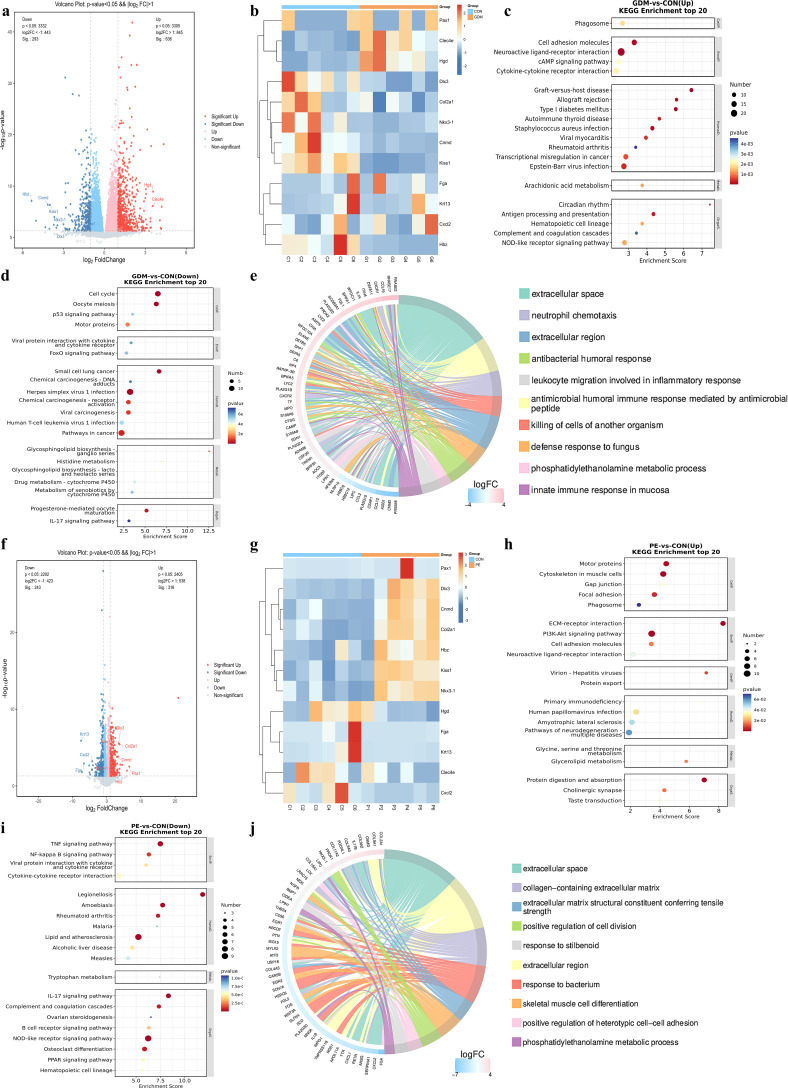
Differential gene expression and functional enrichment in fetal lungs of GDM and PE versus controls. GDM, Gestational Diabetes Mellitus; PE, Preeclampsia; KEGG, Kyoto Encyclopedia of Genes and Genomes. **(a-e)** GDM vs. normal group, **(a)** Volcano plot displaying differentially expressed genes (DEGs) between the GDM and normal groups (*P* < 0.05, |log_2_FC| > 1). Red dots indicate upregulated genes, and blue dots indicate downregulated genes. **(b)** Heatmap of the top DEGs showing expression patterns across samples. **(c, d)** KEGG pathway enrichment analysis of upregulated and downregulated genes in the GDM group (top 20 enriched pathways). **(e)** Gene Ontology (GO) enrichment chord diagram illustrating biological processes significantly enriched among DEGs. **(f-j)** PE vs. normal group. **(f)** Volcano plot showing DEGs between the PE and normal groups. **(g)** Heatmap of the top DEGs in the PE comparison. (h–i) KEGG pathway enrichment analysis of upregulated and downregulated genes in the PE group (top 20 enriched pathways). **(j)** GO enrichment chord diagram demonstrating the significantly enriched biological processes associated with DEGs in PE.

Transcriptomic sequencing was performed to assess gene expression changes in fetal lungs across the normal, GDM, and PE groups, and 12 DEGs (*Hgd*, *Clec4e*, *Nkx3-1*, *Kiss1*, *Cnmd*, *Hbz*, *Fga*, *Krt13*, *Cxcl2*, *Col2a1*, *Pax1*, and *Dlx3*) were selected for further analysis based on their significant expression changes and group-specific patterns (**Supplementary Table 4**, **Figures 7A, B, F-G**). In the GDM group, *Hgd* and *Clec4e* were significantly upregulated, whereas *Nkx3-1*, *Kiss1*, *Cnmd*, and *Hbz* were significantly downregulated compared with those in the normal group. In the PE group, *Pax1*, *Col2a1*, and *Dlx3* were significantly upregulated, whereas *Cxcl2*, *Fga*, and *Krt13* were downregulated.

#### 3.3.2 Functional annotation of DEGs in GDM group

GO enrichment analysis of DEGs in the GDM group identified the top 10 enriched GO terms (**Figure 7E**), including extracellular space, neutrophil chemotaxis, extracellular region, antibacterial humoral response, leukocyte migration involved in inflammatory response, antimicrobial humoral response mediated by antimicrobial peptides, killing of target cells of another organism, defense response to fungus, phosphatidylethanolamine metabolic process, and innate immune response in the mucosa.

Bubble plots illustrating the top 20 KEGG pathways enriched for upregulated and downregulated genes in the GDM group are presented in **Figures 7C, D**, respectively. Enrichment analysis of upregulated genes was mainly associated with cell adhesion molecules, neuroactive ligand-receptor interaction, and phagosome in the environmental information processing category and graft versus host disease in the human diseases category. Conversely, the enriched pathways for downregulated genes were mainly included cell cycle and oocyte meiosis in the cellular processes category and herpes simplex virus 1 infection in the human diseases category.

#### 3.3.3 Functional annotation of DEGs in the PE group

GO enrichment analysis of DEGs in the PE group identified the top 10 enriched GO terms (**Figure 7J**), including the extracellular space, collagen-containing extracellular matrix, extracellular matrix structural constituents conferring tensile strength, positive regulation of cell division, response to bacteria, extracellular region, skeletal muscle cell differentiation, positive regulation of heterotypic cell-cell adhesion, and phosphatidylethanolamine metabolic process.

Bubble plots illustrating the top 20 KEGG pathways enriched for the upregulated and downregulated genes in the PE group are presented in **Figures 7H, I**. The enrichment analysis of the upregulated genes was primarily associated with motor proteins and cytoskeletal organization in muscle cells in the cellular processes category, ECM receptor interaction and the PI3K Akt signaling pathway in the environmental information processing category, and protein digestion and absorption in the organismal systems category. Conversely, the enriched pathways for downregulated genes were mainly included the TNF signaling pathway in the environmental information processing category, legionellosis and lipid and atherosclerosis in the human diseases category, and the NOD-like receptor signaling pathway in the organismal systems category.

### 3.4 Performance of various combination models in assessing fetal lung development in rats

**Figure 8:**
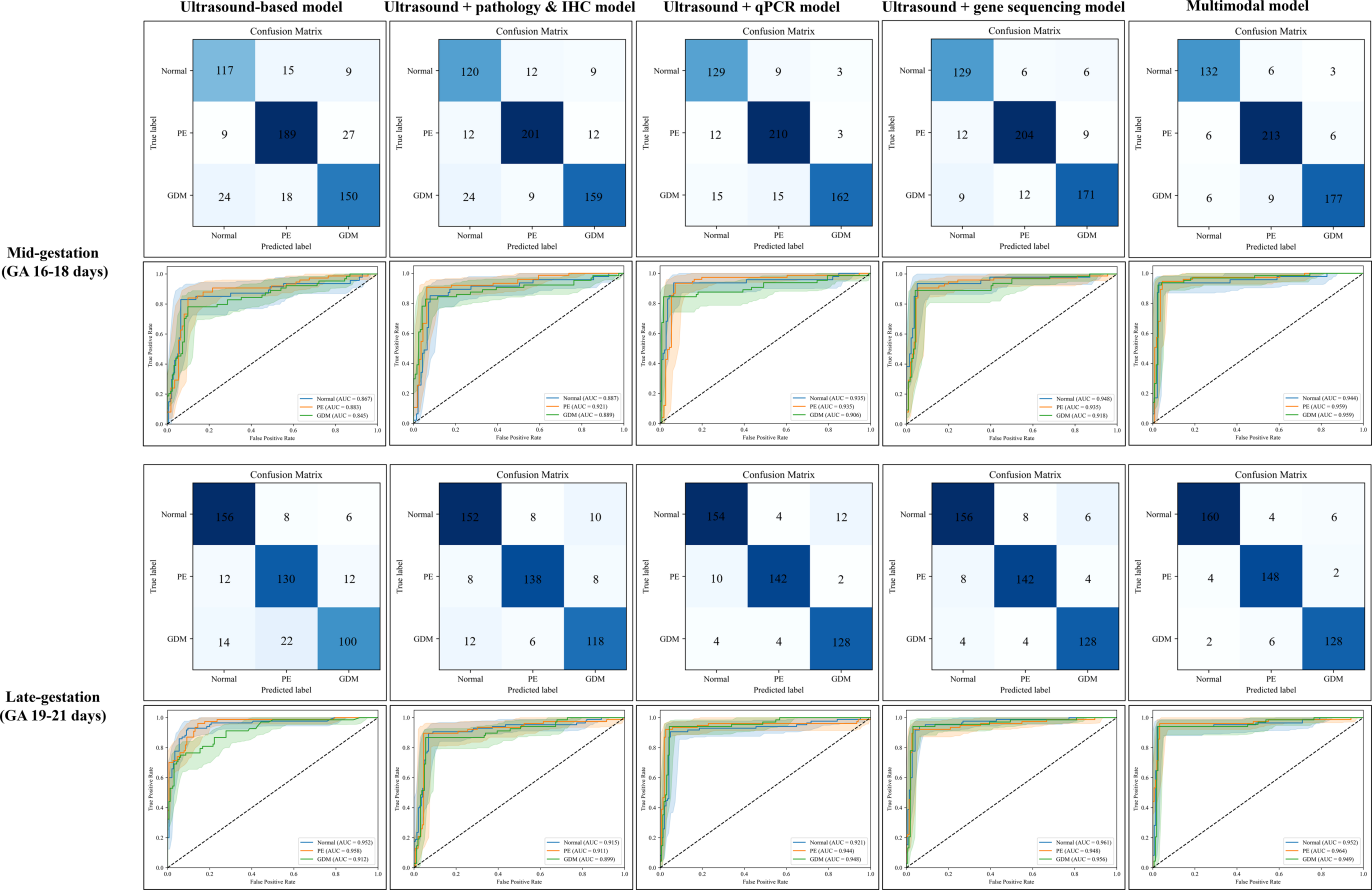
ROC curves and confusion matrices of various combined models. GDM, Gestational Diabetes Mellitus; PE, Preeclampsia; ROC, Receiver Operating Characteristic; AUC, Area Under the Curve; GA, Gestational Age; IHC, Immunohistochemistry. Confusion matrices and ROC curves are shown for models developed using multilevel data at two distinct gestational windows: mid-gestation (GA 16–18 days) and late-gestation (GA 19–21 days). Five models were evaluated: (1) Ultrasound-based model, (2) Ultrasound + Pathology & IHC model, (3) Ultrasound + qPCR model, (4) Ultrasound + gene sequencing model, and (5) Fully integrated multimodal data model. Each model was constructed to differentiate between the normal, GDM, and PE groups. The confusion matrices illustrate the classification accuracy of each model, and the ROC curves provide AUC values that reflect the overall model performance.

A series of ultrasound-based classification models integrating biological information at different levels was developed and compared to evaluate their performance in assessing fetal lung development in rat models of pregnancy complications. The comparison of the diagnostic efficacies of these combination models was shown in **Figure 8**; **Table 4**, **5**.

**Table 4:** Comparison of various combined models for assessing fetal rat lung development in pregnancy complications during mid-gestation.

Diagnostic efficacy		US	US+ Path & IHC	US + qPCR	US + Seq	Multimodal
Accuracy		0.817 [0.763, 0.871]	0.860 [0.820, 0.909]	0.898 [0.857, 0.944]	0.903 [0.860, 0.946]	0.935 [0.898, 0.973]
Average	Sensitivity	0.817 [0.762, 0.870]	0.858 [0.816, 0.905]	0.897 [0.854, 0.947]	0.904 [0.865, 0.945]	0.935 [0.894, 0.977]
	Specificity	0.908 [0.880, 0.935]	0.931 [0.912, 0.956]	0.949 [0.927, 0.972]	0.952 [0.931, 0.974]	0.967 [0.947, 0.988]
	PPV	0.813 [0.755, 0.868]	0.853 [0.813, 0.901]	0.896 [0.858, 0.942]	0.899 [0.858, 0.943]	0.934 [0.889, 0.975]
	NPV	0.907 [0.879, 0.934]	0.930 [0.909, 0.954]	0.949 [0.931, 0.972]	0.951 [0.928, 0.973]	0.967 [0.948, 0.987]
	F1-score	0.814 [0.758, 0.865]	0.854 [0.811, 0.901]	0.895 [0.850, 0.942]	0.901 [0.859, 0.943]	0.934 [0.890, 0.974]
	AUC	0.865 [0.821, 0.904]	0.899 [0.861, 0.939]	0.925 [0.890, 0.964]	0.934 [0.900, 0.966]	0.954 [0.919, 0.984]
Normal	Sensitivity	0.830 [0.734, 0.927]	0.851 [0.760, 0.945]	0.915 [0.826, 0.978]	0.915 [0.837, 0.990]	0.936 [0.871, 1.000]
	Specificity	0.921 [0.887, 0.962]	0.914 [0.856, 0.956]	0.935 [0.907, 0.979]	0.950 [0.919, 0.979]	0.971 [0.943, 1.000]
	PPV	0.780 [0.670, 0.878]	0.769 [0.651, 0.880]	0.827 [0.760, 0.918]	0.860 [0.776, 0.931]	0.917 [0.822, 1.000]
	NPV	0.941 [0.899, 0.974]	0.948 [0.915, 0.981]	0.970 [0.936, 0.993]	0.971 [0.939, 0.997]	0.978 [0.955, 1.000]
	F1-score	0.804 [0.720, 0.887]	0.808 [0.726, 0.878]	0.869 [0.816, 0.929]	0.887 [0.824, 0.937]	0.926 [0.858, 0.979]
	AUC	0.867 [0.804, 0.929]	0.887 [0.815, 0.939]	0.935 [0.882, 0.983]	0.948 [0.901, 0.983]	0.944 [0.899, 0.987]
GDM	Sensitivity	0.781 [0.690, 0.889]	0.828 [0.722, 0.912]	0.844 [0.751, 0.938]	0.891 [0.818, 0.950]	0.922 [0.842, 0.978]
	Specificity	0.902 [0.836, 0.947]	0.943 [0.900, 0.980]	0.984 [0.960, 1.000]	0.959 [0.918, 0.988]	0.975 [0.948, 1.000]
	PPV	0.806 [0.688, 0.897]	0.883 [0.815, 0.955]	0.964 [0.916, 1.000]	0.919 [0.845, 0.977]	0.952 [0.900, 1.000]
	NPV	0.887 [0.826, 0.938]	0.913 [0.865, 0.965]	0.923 [0.874, 0.964]	0.944 [0.902, 0.977]	0.960 [0.924, 0.988]
	F1-score	0.794 [0.708, 0.866]	0.855 [0.795, 0.908]	0.900 [0.837, 0.948]	0.905 [0.851, 0.952]	0.937 [0.882, 0.980]
	AUC	0.845 [0.797, 0.903]	0.889 [0.820, 0.945]	0.906 [0.834, 0.957]	0.918 [0.877, 0.962]	0.959 [0.923, 0.986]
PE	Sensitivity	0.840 [0.744, 0.910]	0.893 [0.824, 0.958]	0.933 [0.884, 0.988]	0.907 [0.831, 0.961]	0.947 [0.891, 0.994]
	Specificity	0.901 [0.845, 0.952]	0.937 [0.890, 0.982]	0.928 [0.873, 0.974]	0.946 [0.900, 0.987]	0.955 [0.919, 0.992]
	PPV	0.851 [0.776, 0.931]	0.905 [0.845, 0.969]	0.897 [0.826, 0.959]	0.919 [0.841, 0.979]	0.934 [0.861, 0.987]
	NPV	0.893 [0.830, 0.939]	0.929 [0.877, 0.973]	0.954 [0.927, 0.991]	0.938 [0.881, 0.974]	0.964 [0.925, 0.996]
	F1-score	0.846 [0.778, 0.904]	0.899 [0.853, 0.954]	0.915 [0.872, 0.959]	0.913 [0.865, 0.967]	0.940 [0.897, 0.982]
	AUC	0.883 [0.817, 0.937]	0.921 [0.884, 0.967]	0.935 [0.895, 0.975]	0.935 [0.896, 0.983]	0.959 [0.925, 0.987]

GDM, Gestational Diabetes Mellitus. PE, Preeclampsia. AUC, Area Under the Curve. PPV, Positive Predictive Value. NPV, Negative Predictive Value. GA, Gestational Age. IHC, Immunohistochemistry.

US, Ultrasound-based model; US + Path & IHC, Ultrasound + Pathology & IHC model; US + qPCR, Ultrasound + qPCR model; US + Seq, Ultrasound + gene sequencing model; Multimodal: Fully integrated multimodal data model (US + Path + IHC + qPCR + Seq). Sensitivity, specificity, PPV, NPV, F1-score and AUC for models are shown with 95% confidence intervals in parentheses.

**Table 5:** Comparison of various combined models for assessing fetal rat lung development in pregnancy complications during late-gestation.

Diagnostic efficacy		US	US + Path & IHC	US + qPCR	US + Seq	Multimodal
Accuracy		0.839 [0.793, 0.883]	0.887 [0.833, 0.924]	0.922 [0.896, 0.959]	0.926 [0.891, 0.954]	0.948 [0.919, 0.970]
Average	Sensitivity	0.832 [0.782, 0.875]	0.886 [0.830, 0.924]	0.923 [0.897, 0.958]	0.927 [0.892, 0.955]	0.948 [0.920, 0.970]
	Specificity	0.919 [0.896, 0.941]	0.943 [0.916, 0.961]	0.961 [0.947, 0.979]	0.963 [0.945, 0.977]	0.974 [0.960, 0.985]
	PPV	0.839 [0.789, 0.884]	0.886 [0.832, 0.923]	0.922 [0.896, 0.958]	0.926 [0.892, 0.954]	0.947 [0.919, 0.971]
	NPV	0.921 [0.899, 0.944]	0.943 [0.916, 0.962]	0.960 [0.947, 0.979]	0.963 [0.945, 0.977]	0.974 [0.960, 0.985]
	F1-score	0.834 [0.784, 0.876]	0.886 [0.829, 0.923]	0.922 [0.896, 0.958]	0.926 [0.892, 0.955]	0.947 [0.918, 0.970]
	AUC	0.941 [0.916, 0.963]	0.908 [0.869, 0.941]	0.938 [0.912, 0.972]	0.955 [0.927, 0.975]	0.955 [0.932, 0.979]
Normal	Sensitivity	0.918 [0.870, 0.975]	0.894 [0.832, 0.957]	0.906 [0.855, 0.972]	0.918 [0.860, 0.976]	0.941 [0.880, 0.987]
	Specificity	0.910 [0.872, 0.946]	0.931 [0.891, 0.972]	0.952 [0.919, 0.982]	0.959 [0.929, 0.990]	0.979 [0.958, 1.000]
	PPV	0.857 [0.803, 0.916]	0.884 [0.828, 0.953]	0.917 [0.871, 0.971]	0.929 [0.883, 0.982]	0.964 [0.927, 1.000]
	NPV	0.950 [0.916, 0.985]	0.938 [0.901, 0.973]	0.945 [0.905, 0.983]	0.952 [0.921, 0.986]	0.966 [0.928, 0.993]
	F1-score	0.886 [0.842, 0.932]	0.889 [0.841, 0.926]	0.911 [0.879, 0.956]	0.923 [0.882, 0.962]	0.952 [0.912, 0.985]
	AUC	0.952 [0.924, 0.978]	0.915 [0.878, 0.954]	0.921 [0.879, 0.969]	0.961 [0.932, 0.986]	0.952 [0.912, 0.987]
GDM	Sensitivity	0.735 [0.608, 0.836]	0.868 [0.793, 0.928]	0.941 [0.888, 0.985]	0.941 [0.883, 0.994]	0.941 [0.884, 0.985]
	Specificity	0.944 [0.911, 0.975]	0.944 [0.905, 0.970]	0.957 [0.930, 0.988]	0.969 [0.934, 0.988]	0.975 [0.952, 0.994]
	PPV	0.847 [0.768, 0.922]	0.868 [0.771, 0.928]	0.901 [0.830, 0.969]	0.928 [0.853, 0.973]	0.941 [0.891, 0.984]
	NPV	0.895 [0.843, 0.933]	0.944 [0.909, 0.970]	0.975 [0.955, 0.994]	0.975 [0.953, 0.997]	0.975 [0.950, 0.994]
	F1-score	0.787 [0.689, 0.851]	0.868 [0.785, 0.913]	0.921 [0.872, 0.960]	0.934 [0.873, 0.972]	0.941 [0.902, 0.969]
	AUC	0.912 [0.864, 0.945]	0.899 [0.841, 0.938]	0.948 [0.923, 0.975]	0.956 [0.916, 0.987]	0.949 [0.908, 0.977]
PE	Sensitivity	0.844 [0.784, 0.909]	0.896 [0.811, 0.964]	0.922 [0.875, 0.973]	0.922 [0.872, 0.976]	0.961 [0.921, 1.000]
	Specificity	0.902 [0.853, 0.950]	0.954 [0.922, 0.984]	0.974 [0.947, 0.997]	0.961 [0.931, 0.993]	0.967 [0.934, 0.987]
	PPV	0.812 [0.708, 0.904]	0.908 [0.836, 0.967]	0.947 [0.890, 0.994]	0.922 [0.861, 0.985]	0.937 [0.884, 0.976]
	NPV	0.920 [0.884, 0.955]	0.948 [0.909, 0.982]	0.961 [0.941, 0.986]	0.961 [0.928, 0.987]	0.980 [0.960, 1.000]
	F1-score	0.828 [0.763, 0.894]	0.902 [0.836, 0.946]	0.934 [0.895, 0.966]	0.922 [0.876, 0.958]	0.949 [0.910, 0.981]
	AUC	0.958 [0.933, 0.980]	0.911 [0.845, 0.953]	0.944 [0.896, 0.985]	0.948 [0.914, 0.982]	0.964 [0.934, 0.988]

GDM, Gestational Diabetes Mellitus. PE, Preeclampsia. AUC, Area Under the Curve. PPV, Positive Predictive Value. NPV, Negative Predictive Value. GA, Gestational Age. IHC, Immunohistochemistry..

US, Ultrasound-based model; US + Path & IHC, Ultrasound + Pathology & IHC model; US + qPCR, Ultrasound + qPCR model; US + Seq, Ultrasound + gene sequencing model; Multimodal: Fully integrated multimodal data model (US + Path + IHC + qPCR + Seq). Sensitivity, specificity, PPV, NPV, F1-score and AUC for models are shown with 95% confidence intervals in parentheses.

Regardless of the gestational age, the models based solely on ultrasound imaging yielded the lowest diagnostic performance in assessing fetal lung development during pregnancy complications (mid-gestation model: accuracy = 0.817 [0.763, 0.871], average AUC = 0.865 [0.821, 0.904]; late-gestation model: accuracy = 0.839 [0.793, 0.883], average AUC = 0.941 [0.916, 0.963]). Conversely, the fully integrated model demonstrated superior diagnostic performance. It combined ultrasound image features with histopathology, IHC, qPCR, and gene sequencing data. The accuracy was 0.935 [0.898, 0.973] (average AUC = 0.954 [0.919, 0.984]) in mid- gestation and 0.948 [0.919, 0.970] (average AUC = 0.955 [0.932, 0.979]) in late- gestation.

### 3.5 Performance of the transfer learning-based model for assessing human fetal lung development

**Figure 9:**
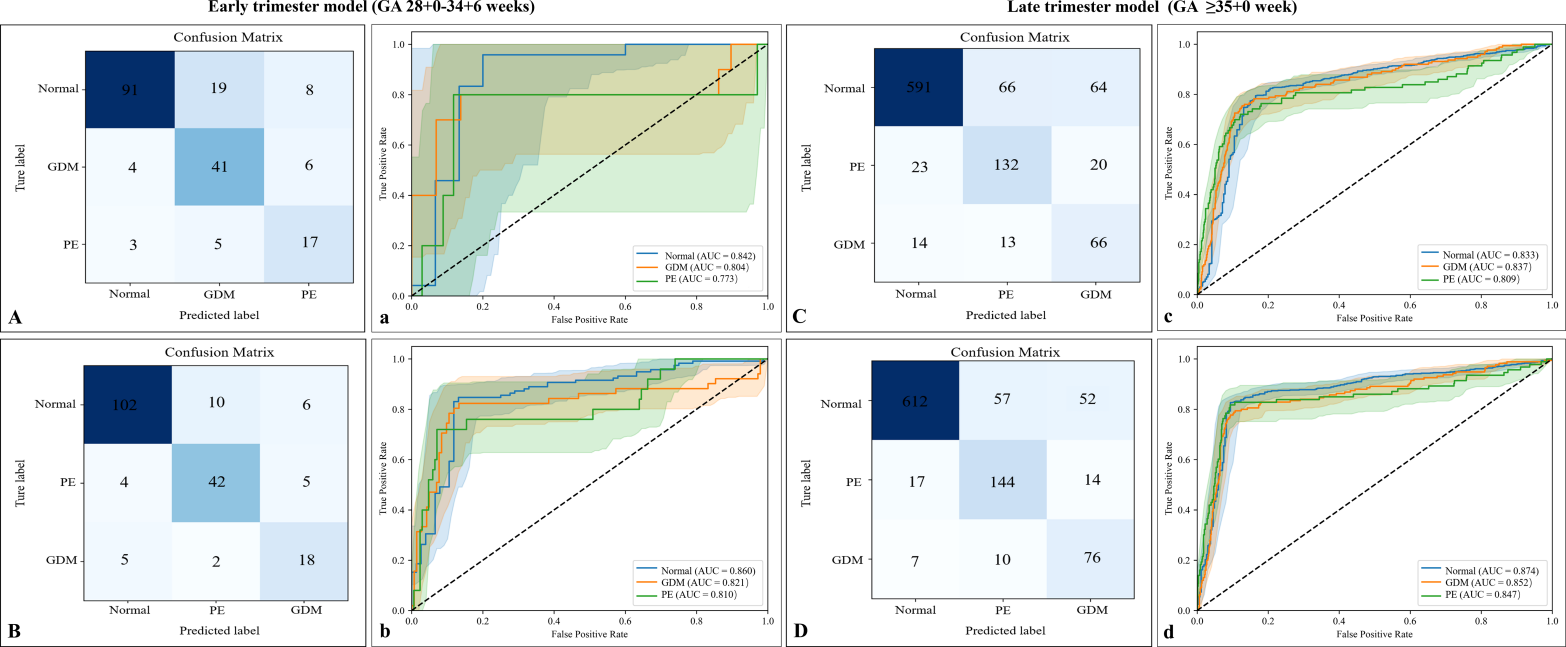
ROC curves and confusion matrices for ultrasound features-based and transfer learning-based models. GDM, Gestational Diabetes Mellitus; PE, Preeclampsia; ROC, Receiver Operating Characteristic; AUC, Area Under the Curve; GA, Gestational Age. **(A-D)** depict the confusion matrices, and a-d display the corresponding ROC curves. A/a and C/c illustrate the performance of ultrasound features-based models for classifying fetal lung development in human pregnancy complications. B/b and D/d represent the performances of the transfer learning-based classification models.

The diagnostic performance of the human fetal lung classification model was evaluated. The model was developed using transfer learning based on a multilevel model from rat data. It was compared with a model built only from human fetal lung ultrasound images (**Table 6**; **Figure 9**). In both early and late trimester analyses, transfer learning-based human models showed better performance compared with ultrasound-only models. The early trimester model yielded an accuracy of 0.835 [0.786, 0.894] and an average AUC of 0.830 [0.772, 0.890], whereas the late trimester model achieved an accuracy of 0.874 [0.828, 0.907] and an average AUC of 0.857 [0.824, 0.893].

**Table 6:** Comparison of ultrasound features-based and transfer learning-based models for assessing fetal lung development in pregnancy complications.

Model	Accuracy		Sensitivity	Specificity	PPV	NPV	F1-score	AUC
US -early	0.768 [0.709, 0.825]	Average	0.752 [0.682, 0.821]	0.886 [0.852, 0.912]	0.703 [0.628, 0.776]	0.864 [0.832, 0.898]	0.719 [0.642, 0.787]	0.806 [0.747, 0.869]
		Normal	0.771 [0.688, 0.838]	0.908 [0.857, 0.971]	0.929 [0.885, 0.979]	0.719 [0.630, 0.802]	0.843 [0.775, 0.886]	0.842 [0.784, 0.895]
		GDM	0.804 [0.672, 0.898]	0.832 [0.761, 0.890]	0.631 [0.529, 0.775]	0.922 [0.869, 0.961]	0.707 [0.606, 0.813]	0.804 [0.710, 0.879]
		PE	0.680 [0.471, 0.854]	0.917 [0.876, 0.954]	0.548 [0.365, 0.705]	0.951 [0.918, 0.982]	0.607 [0.455, 0.734]	0.773 [0.655, 0.891]
TL-Early	0.835 [0.786, 0.894]	Average	0.803 [0.736, 0.881]	0.911 [0.885, 0.944]	0.772 [0.711, 0.847]	0.900 [0.872, 0.934]	0.786 [0.721, 0.860]	0.830 [0.772, 0.890]
		Normal	0.864 [0.794, 0.911]	0.882 [0.829, 0.956]	0.919 [0.877, 0.969]	0.807 [0.726, 0.876]	0.891 [0.844, 0.930]	0.860 [0.808, 0.914]
		GDM	0.824 [0.732, 0.930]	0.916 [0.850, 0.954]	0.778 [0.649, 0.872]	0.936 [0.903, 0.975]	0.800 [0.708, 0.892]	0.821 [0.748, 0.906]
		PE	0.720 [0.558, 0.905]	0.935 [0.904, 0.970]	0.621 [0.469, 0.770]	0.958 [0.928, 0.988]	0.667 [0.521, 0.810]	0.810 [0.720, 0.910]
US-late	0.798 [0.770, 0.820]	Average	0.761 [0.724, 0.795]	0.890 [0.876, 0.905]	0.669 [0.631, 0.701]	0.851 [0.834, 0.865]	0.701 [0.660, 0.735]	0.826 [0.793, 0.849]
		Normal	0.820 [0.793, 0.843]	0.862 [0.819, 0.897]	0.941 [0.925, 0.956]	0.640 [0.590, 0.682]	0.876 [0.861, 0.893]	0.833 [0.800, 0.854]
		GDM	0.754 [0.687, 0.818]	0.903 [0.881, 0.919]	0.626 [0.562, 0.677]	0.945 [0.929, 0.959]	0.684 [0.616, 0.730]	0.837 [0.796, 0.865]
		PE	0.710 [0.629, 0.792]	0.906 [0.886, 0.922]	0.440 [0.371, 0.513]	0.968 [0.957, 0.978]	0.543 [0.479, 0.611]	0.809 [0.750, 0.860]
TL-Late	0.874 [0.828, 0.907]	Average	0.858 [0.834, 0.883]	0.830 [0.798, 0.865]	0.918 [0.906, 0.930]	0.727 [0.692, 0.770]	0.877 [0.862, 0.896]	0.857 [0.824, 0.893]
		Normal	0.849 [0.823, 0.874]	0.910 [0.879, 0.942]	0.962 [0.948, 0.974]	0.691 [0.644, 0.741]	0.902 [0.888, 0.919]	0.874 [0.848, 0.896]
		GDM	0.823 [0.789, 0.871]	0.918 [0.898, 0.937]	0.682 [0.619, 0.746]	0.960 [0.951, 0.972]	0.746 [0.702, 0.799]	0.852 [0.829, 0.887]
		PE	0.817 [0.741, 0.892]	0.926 [0.909, 0.943]	0.535 [0.459, 0.620]	0.980 [0.972, 0.989]	0.647 [0.574, 0.713]	0.847 [0.796, 0.895]

GDM, Gestational Diabetes Mellitus; PE, Preeclampsia; AUC, Area Under the Curve; PPV, Positive Predictive Value; NPV, Negative Predictive Value; GA, Gestational Age.

US models: Developed using human fetal lung ultrasound images, with the early model trained on scans acquired at 28^+0^- 34^+6^ weeks’ gestation and the late model on scans acquired at ≥35^+0^ weeks. TL models: Generated by transfer learning from rat multimodal models and subsequently fine-tuned with human ultrasound images, using the same gestational age ranges for the early (28^+0^- 34^+6^ weeks) and late (≥35^+0^ weeks) trimester models.

## 4 Discussion

This study proposed a cross-species multimodal data-integrated model for characterizing fetal lung development in normal and complicated pregnancies. In animal studies, the integrated multimodal model demonstrated superior performance in discriminating fetal lung development among GDM, PE, and normal groups compared with unimodal approaches, which combined ultrasound imaging features, lung histopathology, targeted molecular expression, and transcriptomic analyses. Using transfer learning methods, this comprehensive framework was validated in human pregnancy data, and it can support future studies on the use of this non-invasive approach for more accurate management of high-risk pregnancies.

GDM and PE are known to affect fetal lung development through different pathological mechanisms (33, 34). In this study, compared with the normal group, newborn rats in the GDM group showed increased body weight but reduced lung weight, and this finding was consistent with the disproportionate somatic growth reported in diabetic pregnancy (35). Molecular analysis showed increased expression of surfactant proteins (SP-A, SP-B, SP-C, and SP-D) and VEGF in fetal lungs exposed to GDM. These results suggested that early or abnormal activation of lung maturation pathways in GDM reflects compensatory responses to metabolic stress and an inflammatory intrauterine environment, rather than normal and coordinated lung maturation. This interpretation is also supported by previous studies that described metabolic stress, chronic low-grade inflammation, and altered growth signaling in GDM pregnancies (36–38).

Transcriptomic analysis further supported these findings and showed widespread gene expression changes related to metabolism, immune activation, vascular remodeling, and tissue organization in fetal lungs exposed to GDM. GO enrichment analysis highlighted extracellular matrix remodeling, immune cell chemotaxis, and innate immune responses, and this suggested the presence of a pro-inflammatory intrauterine environment. The increased expression of the innate immune signaling gene *Clec4e* was consistent with enrichment of leukocyte migration and antibacterial response pathways, and it supported the activation of intrauterine inflammation (39, 40). Increased expression of *Hgd* may reflect changes in tyrosine metabolism and metabolic instability, which is consistent with reports of oxidative stress and amino acid imbalance in diabetic pregnancy (41, 42). In contrast, reduced expression of genes involved in cell cycle control and epithelial differentiation, including *Nkx3–1* and *Kiss1*, indicated impaired cell proliferation and endocrine signaling, which may further disrupt coordinated lung development (43, 44).

In the PE model, fetal lung maturation was delayed compared with the normal group. Both body weight and lung weight of newborn rats exposed to PE were significantly reduced, and histological analysis showed features of arrested tubular development at the canalicular stage, including thickened alveolar septa, narrowed air spaces, and reduced airway walls thickness (25). Pulmonary artery thickening was also observed, and this finding suggested early vascular remodeling that may increase the risk of respiratory dysfunction after birth (45). Molecular analysis showed a general decrease in SP and VEGF expression in the PE group, which is consistent with previous studies reporting impaired alveolarization and angiogenesis in PE pregnancies (46, 47).

Transcriptome analysis of fetal lungs exposed to PE showed coordinated changes in genes related to extracellular matrix (ECM) organization, mesenchymal activity, and growth regulation. Enrichment of ECM remodeling, cytoskeletal regulation, and the PI3K-Akt signaling pathway suggested enhanced mesenchymal ECM signaling and altered epithelial-mesenchymal interactions (48–50). Increased expression of regulatory genes such as *Pax1*, *Col2a1*, and *Dlx3* further promoted mesenchymal activity, and this may lead to membrane thickening and reduced alveolarization (51–53). In contrast, downregulated genes were mainly involved in immune and inflammatory signaling pathways, including TNF and NOD-like receptor signaling (54, 55). Reduced expression of *Cxcl2* and *Fga* indicated weakened innate immune and coagulation responses, which may interfere with inflammatory signaling required for normal lung maturation (56).

With the increasing understanding of disease-related molecular complexity, many studies have combined imaging data with molecular and genomic information to improve diagnostic accuracy and biological interpretation (19, 57–59). In this study, animal experiments showed that models combining multimodal data performed better than ultrasound-only models in classifying fetal lung development under pregnancy complications, and these results were also validated in human data. This finding is consistent with the study by Min Hu et al., which highlighted the value of integrating multiple data sources to improve predictive performance (60).

Because direct intrauterine assessment of fetal lung development is difficult in clinical practice, we performed a cross-species study to address this problem. In this study, a multimodal data fusion rat fetal lung development model was applied to human fetal samples through transfer learning methods, attempting to provide a biological basis for evaluating human fetal lung development. Transfer learning is considered a powerful tool that bridges the gap between data domains by transferring knowledge from the source domain to the target domain (61), and has been shown to significantly improve model performance when adapting to new tasks or transferring datasets across different institutions (62, 63). This deep learning framework may provide a foundation for non-invasive identification of high-risk fetuses in the future and support more accurate fetal management.

Our research has some limitations. Firstly, although the transfer learning strategy from animals to humans has addressed the lack of molecular annotation in human fetal lungs, interspecies differences in lung development and gene regulation limit direct extrapolation of molecular features. Secondly, the relationship between prenatal assessment and neonatal respiratory outcomes was not examined. Because of this, the clinical use of the model is limited. The model was not tested with real neonatal outcomes, so it cannot be used directly in clinical decision-making. In the future, prospective multicenter studies are necessary. Patients should be followed over time, and prenatal ultrasound data should be linked with neonatal respiratory outcomes. Thirdly, in the animal experiments, differences between fetuses from the same dam, together with the incomplete one-to-one matching between imaging and molecular samples, may introduce a certain degree of heterogeneity, which should be taken into consideration when interpreting the findings. Finally, although the model exhibits robustness, it is hard to understand how the model makes predictions and determine which parts of the fetal lung images are most important. In the future, explainable methods such as Grad-CAM and attention map visualization can be used. These methods can highlight important regions in the images and show which areas drive the model predictions. This can help improve model transparency and make the results easier to understand.

## 5 Conclusions

In summary, this study showed the benefit of combining multimodal data to assess fetal lung development during GDM and PE. The proposed approach showed stable and reliable performance and was validated using clinical data. These results may provide a methodological and biological support for future research on fetal lung development and may help promote the use of this non-invasive framework in the management of high-risk pregnancies.

## Data Availability

The datasets presented in this study can be found in online repositories. The names of therepository/repositories and accession number(s) can be found below: https://doi.org/10.5281/zenodo.15845525,Zenodo 15845525 https://doi.org/10.5281/zenodo.17875951, Zenodo 17875951. The custom Python code used to develop the multimodal and transfer-learning pipelines in this study is publicly available at: https://github.com/BJAICoding/MMDL4FetalLungAna. The repository includes the core scripts required to reproduce the main analyses and model development procedures described in this work.
