# Supplementary material for: Multimodal deep learning fusion model for assessment of fetal lung development in gestational diabetes mellitus and pre-eclampsia
Source: Front Endocrinol (Lausanne). 2026 May 18;17:1832468. doi: 10.3389/fendo.2026.1832468 (PMC13223097; doi:10.3389/fendo.2026.1832468)
Supplement: Supplementary file 1 [file Table1.docx]

**Supplementary Material**

**Table S1 The primer sequences of the interested genes in this study**

| Gene name |  | Primer sequences |
| --- | --- | --- |
| Rat VEGF | Forward | GTCGGAGAGCAACGTCACTA |
|  | Reverse | TGCGCTTTCGTTTTTGACCC |
| Rat SP-A | Forward | TAAGTGCTGCCCTCTGACCT |
|  | Reverse | AGGAGCCATACATGCCAAAC |
| Rat SP-B | Forward | AGCCTGGAGCAAGCGATAC |
|  | Reverse | AAGCGTCTTCCTTGGTCATC |
| Rat SP-C | Forward | CCTTGTCGTCGTGGTGAT |
|  | Reverse | AGGTAGCGATGGTGTCTGT |
| Rat SP-D | Forward | CAGGAAGCAATCTGCCATGC |
|  | Reverse | TCTCTGCGAGAGGGTCTTCA |
| Rat β-actin | Forward | ATGCCATCCTGCGTCTGGA |
|  | Reverse | CACATCTGCTGGAAGGTGG |

SP: Surfactant Protein; VEGF: Vascular Endothelial Growth Factor.

**Table S2 Immunohistochemistry results of fetal lungs**

| **Characteristics** | | **Normal Group** | **GDM Group** | **PE Group** | ***P* Value** |
| --- | --- | --- | --- | --- | --- |
| **SP-A** | **Mean(OD)** | 0.191-0.337  (0.248±0.041) | 0.174-0.290  (0.247±0.0386) | 0.171-0.222  (0.194±0.0117) | *P^1^*=0.158, *P*^2^<0.001^*^, *P*^3^<0.001^*^ |
|  | **%Area** | 1.528-5.743  (3.324±1.414) | 0.536-10.610  (5.183±2.875) | 2.180-5.895  (4.485±1.023) | *P^1^*=0.037^*^, *P*^2^=0.007^*^, *P*^3^=0.250 |
| **SP-B** | **Mean(OD)** | 0.141-0.228  (0.185±0.025) | 0.182-0.325  (0.243±0.034) | 0.129-0.249  (0.179±0.031) | *P*^1^<0.001^*^, *P*^2^=0.555, *P*^3^<0.001^*^ |
|  | **%Area** | 1.032-7.197  (3.976± 2.076) | 2.148-7.807  (4.192±1.766) | 0.979-8.370  (3.895±1.844) | *P*^1^=0.733, *P*^2^=0.883, *P*^3^=0.580 |
| **SP-C** | **Mean(OD)** | 0.217-0.291  (0.258±0.025) | 0.196-0.344  (0.284±0.040) | 0.190-0.363  (0.249±0.039) | *P^1^*=0.042^*^, *P*^2^=0.487, *P*^3^=0.000^*^ |
|  | **%Area** | 2.116-13.905  (8.163±4.020) | 5.534-15.945  (10.459±2.655) | 2.286-11.272  (8.036±2.047) | *P^1^*=0.034^*^, *P*^2^=0.889, *P*^3^=0.000^*^ |
| **SP-D** | **Mean(OD)** | 0.217-0.361  (0.261±0.047) | 0.178-0.437  (0.293±0.060) | 0.171-0.327  (0.258±0.033) | *P^1^*=0.107, *P*^2^=0.784, *P*^3^=0.003^*^ |
|  | **%Area** | 2.195-13.499  (7.558±3.350) | 3.446-13.437  (8.161±2.331) | 2.202-12.974  (7.396±2.352) | *P^1^*=0.465, *P*^2^=0.839, *P*^3^=0.204 |
| **VEGF** | **Mean(OD)** | 0.206-0.454  (0.366±0.078) | 0.274-0.484  (0.407±0.048) | 0.289-0.413  (0.352±0.027) | *P^1^*=0.037^*^, *P*^2^=0.356, *P*^3^<0.001^*^ |
|  | **%Area** | 3.281-15.164  (8.395±2.720) | 2.694-13.708  (10.145±2.616) | 2.641-12.217  (8.244±2.903) | *P*^1^=0.033^*^, *P*^2^=0.865, *P*^3^=0.012^*^ |

GDM: Gestational Diabetes Mellitus; PE: Preeclampsia; SP-A: Surfactant Protein A; SP-B: Surfactant Protein B; SP-C: Surfactant Protein C; SP-D: Surfactant Protein D; VEGF: Vascular Endothelial Growth Factor.

*P*^1^ value is the comparation between normal group and GDM group, *P*^2^ value is the comparation between normal group and PE group, *P*^3^ value is the comparation between GDM group and PE group. ^*^ *P* value < 0.05 indicated a significant difference.

**Table S3** **Comparative qPCR analysis of SP-A, SP-B, SP-C, SP-D, VEGF in normal, GDM, and PE groups**

| **mRNA** | **Normal Group** | **GDM Group** | **PE Group** | ***P* Value** |
| --- | --- | --- | --- | --- |
| **SP-A** | 0.121-13.785  (4.280±5.279) | 0.645-24.451（15.001±7.477） | 1.758-5.549  (3.367±1.034） | *P*^1^=0.001^*^, *P*^2^=0.478, *P*^3^<0.001^*^ |
| **SP-B** | 0.094-19.635  (6.144±7.8524) | 0.379-22.544 (14.203±6.755) | 1.798-5.905  (3.830±1.168) | *P*^1^=0.010^*^, *P*^2^=0.225, *P*^3^<0.001^*^ |
| **SP-C** | 0.089-7.051  (2.587±2.836) | 0.628-20.557 (10.887±5.412) | 0.677-3.129  (1.999±0.540) | *P*^1^=0.000^*^, *P*^2^=0.395, *P*^3^<0.001^*^ |
| **SP-D** | 0.154-20.403  (6.849±8.659) | 0.424-38.201  (23.587±11.254) | 3.187-16.226  (7.239±4.109) | *P*^1^=0.001^*^, *P*^2^=0.872, *P*^3^<0.001^*^ |
| **VEGF** | 0.100-7.349  (2.664±3.068) | 1.283-12.824  (6.403±3.596) | 0.236-2.397  (1.249±0.527) | *P*^1^=0.012^*^, *P*^2^=0.064, *P*^3^<0.001^*^ |

GDM: Gestational Diabetes Mellitus; PE: Preeclampsia; SP-A: Surfactant Protein A; SP-B: Surfactant Protein B; SP-C: Surfactant Protein C; SP-D: Surfactant Protein D; VEGF: Vascular Endothelial Growth Factor.

*P*^1^ value is the comparation between normal group and GDM group, *P*^2^ value is the comparation between normal group and PE group, *P*^3^ value is the comparation between GDM group and PE group. ^*^ *P* value < 0.05 indicated a significant difference.

**Table S4 Differentially expressed genes and functional enrichment in GDM and PE compared to normal group**

| **Groups** | **Gene id** | **Fold change** | **log2FoldChange** | **p-value** | **q-value** | **Regulation direction** | **Description** |
| --- | --- | --- | --- | --- | --- | --- | --- |
| **GDM group**  **vs**  **Normal group** | *Hgd* | 11.907 | 3.574 | 1.211 × 10⁻⁹ | 5.139× 10⁻^8^ | Up | homogentisate 1%2C 2-dioxygenase |
|  | *Clec4e* | 11.695 | 3.548 | 7.472× 10⁻^7^ | 1.324× 10⁻^5^ | Up | C- type lectin domain family 4%2C member E |
|  | *Nkx3-1* | 0.126 | -2.992 | 0.003 | 0.012 | Down | NK3 homeobox 1 |
|  | *Kiss1* | 0.063 | -3.986 | 4.662× 10⁻^7^ | 8.854× 10⁻^6^ | Down | KiSS-1 metastasis-suppressor |
|  | *Cnmd* | 0.057 | -4.139 | 4.102× 10⁻^7^ | 7.947× 10⁻^6^ | Down | chondromodulin |
|  | *Hbz* | 0.025 | -5.328 | 7.420× 10⁻^8^ | 1.845× 10⁻^6^ | Down | hemoglobin subunit zeta |
| **PE group**  **vs**  **Normal group** | *Pax1* | 95.744 | 6.581 | 0.003 | 0.029 | Up | paired box 1 |
|  | *Col2a1* | 13.241 | 3.727 | 3.746× 10⁻^6^ | 0.000 | Up | collagen type II alpha 1 chain |
|  | *Dlx3* | 11.172 | 3.482 | 2.845× 10⁻^6^ | 0.000 | Up | distal-less homeobox 3 |
|  | *Cxcl2* | 0.053 | -4.247 | 0.001 | 0.011 | Down | C-X-C motif chemokine ligand 2 |
|  | *Fga* | 0.010 | -6.685 | 0.001 | 0.017 | Down | fibrinogen alpha chain |
|  | *Krt13* | 0.005 | -7.523 | 1.312× 10⁻^6^ | 0.000 | Down | keratin 13 |

GDM: Gestational Diabetes Mellitus; PE: Preeclampsia.
